# Inhibition of C1-Ten PTPase activity reduces insulin resistance through IRS-1 and AMPK pathways

**DOI:** 10.1038/s41598-017-18081-8

**Published:** 2017-12-19

**Authors:** Heeyoon Jeong, Ara Koh, Jiyoun Lee, Dohyun Park, Jung Ok Lee, Mi Nam Lee, Kyung-Jin Jo, Huynh Nguyen Khanh Tran, Eui Kim, Byung-Sun Min, Hyeon Soo Kim, Per-Olof Berggren, Sung Ho Ryu

**Affiliations:** 1 Department of Life Sciences, Pohang University of Science and Technology, Pohang, 37673 Republic of Korea; 2 Department of Anatomy, Korea University College of Medicine, Seoul, 02841 Republic of Korea; 3 College of Pharmacy, Drug Research and Development Center, Catholic University of Daegu, Gyeongbuk, 38430 Republic of Korea; 4 Division of Integrative Biosciences and Biotechnology, Pohang University of Science and Technology, Pohang, 37673 Republic of Korea; 5 The Rolf Luft Research Center for Diabetes and Endocrinology, Karolinska Institutet, Karolinska University Hospital, S-171 76 Stockholm, Sweden

## Abstract

Insulin resistance causes type 2 diabetes; therefore, increasing insulin sensitivity is a therapeutic approach against type 2 diabetes. Activating AMP-activated protein kinase (AMPK) is an effective approach for treating diabetes, and reduced insulin receptor substrate-1 (IRS-1) protein levels have been suggested as a molecular mechanism causing insulin resistance. Thus, dual targeting of AMPK and IRS-1 might provide an ideal way to treat diabetes. We found that 15,16-dihydrotanshinone I (DHTS), as a C1-Ten protein tyrosine phosphatase inhibitor, increased IRS-1 stability, improved glucose tolerance and reduced muscle atrophy. Identification of DHTS as a C1-Ten inhibitor revealed a new function of C1-Ten in AMPK inhibition, possibly through regulation of IRS-1. These findings suggest that C1-Ten inhibition by DHTS could provide a novel therapeutic strategy for insulin resistance-associated metabolic syndrome through dual targeting of IRS-1 and AMPK.

## Introduction

Type 2 diabetes is the most common form of diabetes, and insulin resistance in metabolic organs contributes to the development of this disease. Tyrosine phosphorylation of insulin receptor substrate-1 (IRS-1) activates signaling that is important for mediating the metabolic action of insulin by recruiting Src homology 2 (SH2) domain-containing molecules, such as phosphatidylinositol 3-kinase (PI3K). Thus, the main causes of insulin resistance are thought to include significant decreases in IRS-1 protein levels, insulin-stimulated IRS-1 tyrosine phosphorylation, and IRS-1-associated PI3K activity observed in subjects with insulin resistance and type 2 diabetes^1–5^.

Skeletal muscle insulin resistance is considered a primary or initiating defect in type 2 diabetes^6^. Reduced IRS-1 levels contribute to two pathological features of insulin resistance in skeletal muscle: impaired glucose metabolism and decreased muscle mass^7^. Several E3 ligases that target IRS-1 degradation have been implicated in triggering insulin resistance and related metabolic disorders^8–11^. Overexpression of E3 ligases targeting IRS-1 induces pathological changes such as glucose intolerance or muscle atrophy^8–11^, as both are under the control of IRS-1-mediated signaling. To date, no specific inhibitors for increasing IRS-1 protein stability have been reported. Previously, we revealed that C1-Ten promotes IRS-1 degradation via its protein tyrosine phosphatase (PTPase) activity towards IRS-1, thereby reducing glucose uptake and muscle mass^3^. Since C1-Ten is upregulated under insulin-resistant and diabetic conditions (i.e., dexamethasone and rodent diabetic models), a chemical inhibitor for C1-Ten PTPase activity might provide a potential therapeutic approach against insulin resistance and type 2 diabetes.

In this study, we demonstrated that 15,16-dihydrotanshinone I (DHTS), a component of *Salvia miltiorrhiza* (danshen), functions as a C1-Ten inhibitor. We found that DHTS administration improved glucose tolerance and reduced muscle atrophy in the skeletal muscle of diabetic mice (db/db). To determine the underlying mechanism, we demonstrated that DHTS increases IRS-1 protein levels via C1-Ten inhibition. Furthermore, with the unexpected finding that C1-Ten inhibition leads to activation of both IRS-1 and AMP-activated protein kinase (AMPK) pathways, we elucidated a new C1-Ten-mediated signaling pathway using the C1-Ten inhibitor, DHTS. Our study potentiates the importance of C1-Ten as a novel therapeutic target for metabolic disorders.

## Results

### Identification of DHTS as an inhibitor of C1-Ten PTPase

**Figure 1:**
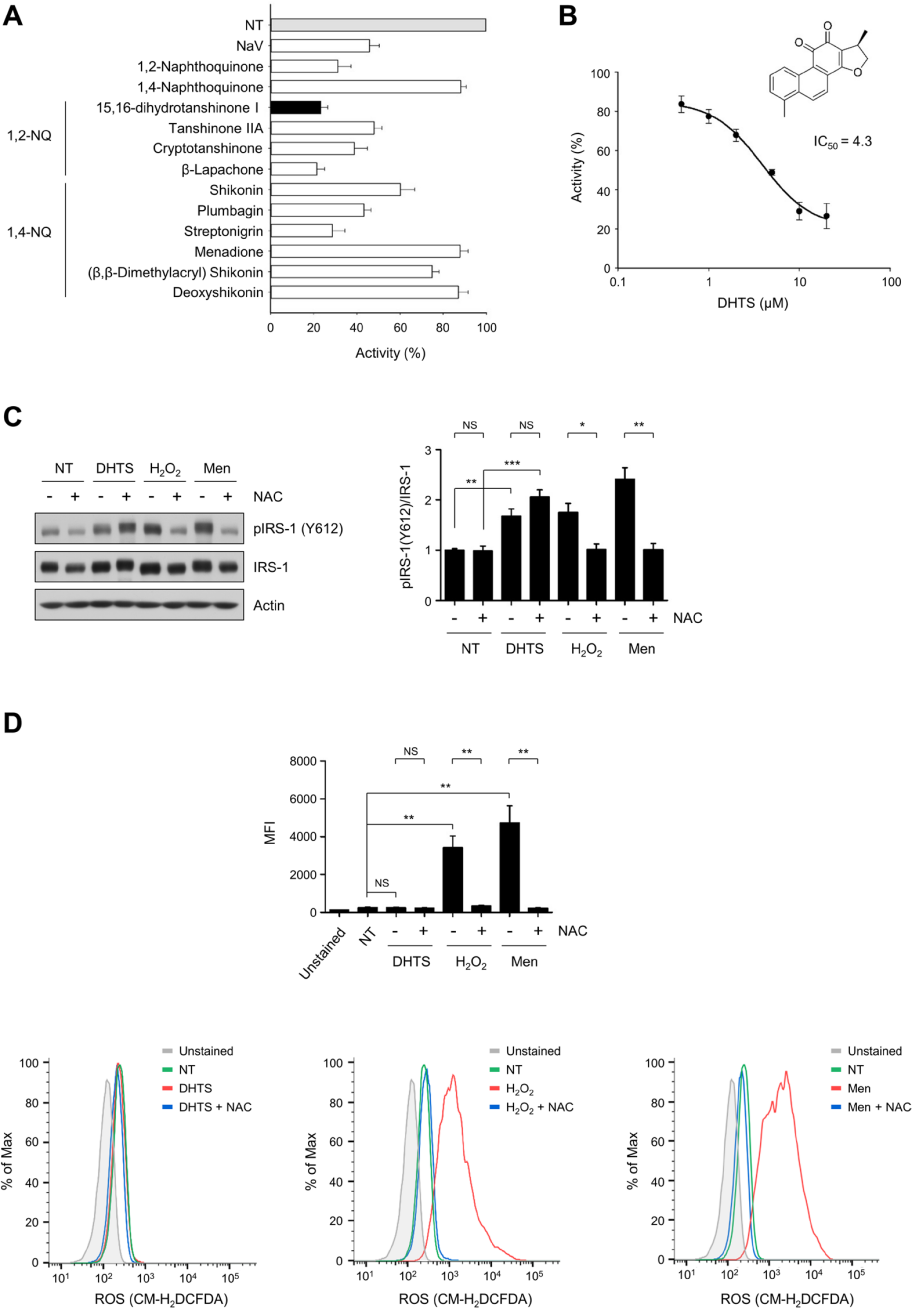
15,16-Dihydrotanshinone I (DHTS) inhibits C1-Ten protein tyrosine phosphatase (PTPase) activity. (**A**) C1-Ten PTPase inhibitor screening. The malachite green assay was performed with 1.5 μg purified C1-Ten protein in the presence of DMSO (NT) or indicated compound (10 μM). Vanadate (NaV) was used as a positive control. Assays were repeated three times in duplicate. Data are presented as the mean ± standard error of the mean (SEM). (**B**) DHTS inhibits C1-Ten PTPase activity with an IC_50_ of 4.3 µM. Assays were repeated three times in duplicate. Data are presented as the mean ± SEM. (**C**) L6 myoblasts were pre-treated with 5 mM N-acetyl-L-cysteine (NAC) for 30 min, and the cells were co-treated with NAC and the indicated compound (DMSO, 10 μM DHTS, 1 mM H_2_O_2_, or 25 μM Menadione) for 1 h. NT, DMSO control. Data are presented as the mean ± SEM (n = 4); *P < 0.05, **P < 0.01, and ***P < 0.001; NS, not significant. (**D**) L6 myoblasts were pre-treated with 5 mM NAC for 30 min, and the cells were co-treated with NAC and the indicated compound (DMSO, 10 μM DHTS, 1 mM H_2_O_2_, or 25 μM Menadione) for 1 h. NT, DMSO control. To determine intracellular ROS generation, the cells were loaded with 5 μM CM-H_2_DCFDA for 30 min. After trypsinization, the fluorescence intensity was measured by flow cytometry. Data are presented as the mean ± SEM (n = 4); **P < 0.01; NS, not significant.

To identify chemical inhibitors for C1-Ten PTPase, we employed an *in vitro* screening system. Active C1-Ten protein was purified from stable FresStyle293-F cells expressing 3 × FLAG tagged-C1-Ten (full-length, wild-type C1-Ten; Supplementary Fig. S1A). Next, we screened a natural product library (502 compounds) for their ability to inhibit C1-Ten. Seven compounds — 15,16-dihydrotanshinone I (DHTS), tanshinone IIA, cryptotanshinone, β-lapachone, shikonin, plumbagin, and streptonigrin — showed inhibitory effects against C1-Ten PTPase activity (Fig. 1A). Interestingly, all of these compounds are naphthoquinone derivatives, with 1,2-naphthoquinone derivatives exhibiting more effective inhibition of C1-Ten PTPase activity than 1,4-naphthoquinone (Fig. 1A, Supplementary Fig. S1B). As DHTS showed the most potent inhibitory effect, the compound was selected for further study. The IC_50_ value of DHTS was 4.3 µM (Fig. 1B). PTPases are readily inactivated by reactive oxygen species (ROS) through the oxidation of catalytic cysteine^12^. To investigate whether DHTS inhibits C1-Ten through ROS generation, we cotreated L6 myoblasts with N-acetyl-L-cysteine (NAC) and DHTS (Fig. 1C). Both ROS-generating agents (H_2_O_2_ and Menadione) and DHTS strongly induced IRS-1 tyrosine phosphorylation. Although NAC treatment prevented H_2_O_2_ and Menadione-induced IRS-1 tyrosine phosphorylation, DHTS-induced IRS-1 tyrosine phosphorylation remained in the presence of NAC. Next, we measured the intracellular ROS levels in cells using flow cytometry (Fig. 1D). DHTS did not generate ROS, excluding the possibility of ROS involvement in DHTS-mediated C1-Ten inhibition. Instead, we observed that DHTS inhibited the interaction between C1-Ten and IRS-1 (Supplementary Fig. S1C). Therefore, we conclude that DHTS inhibits C1-Ten PTPase activity in a ROS-independent manner.

### DHTS improves diabetic phenotypes in db/db mice

**Figure 2:**
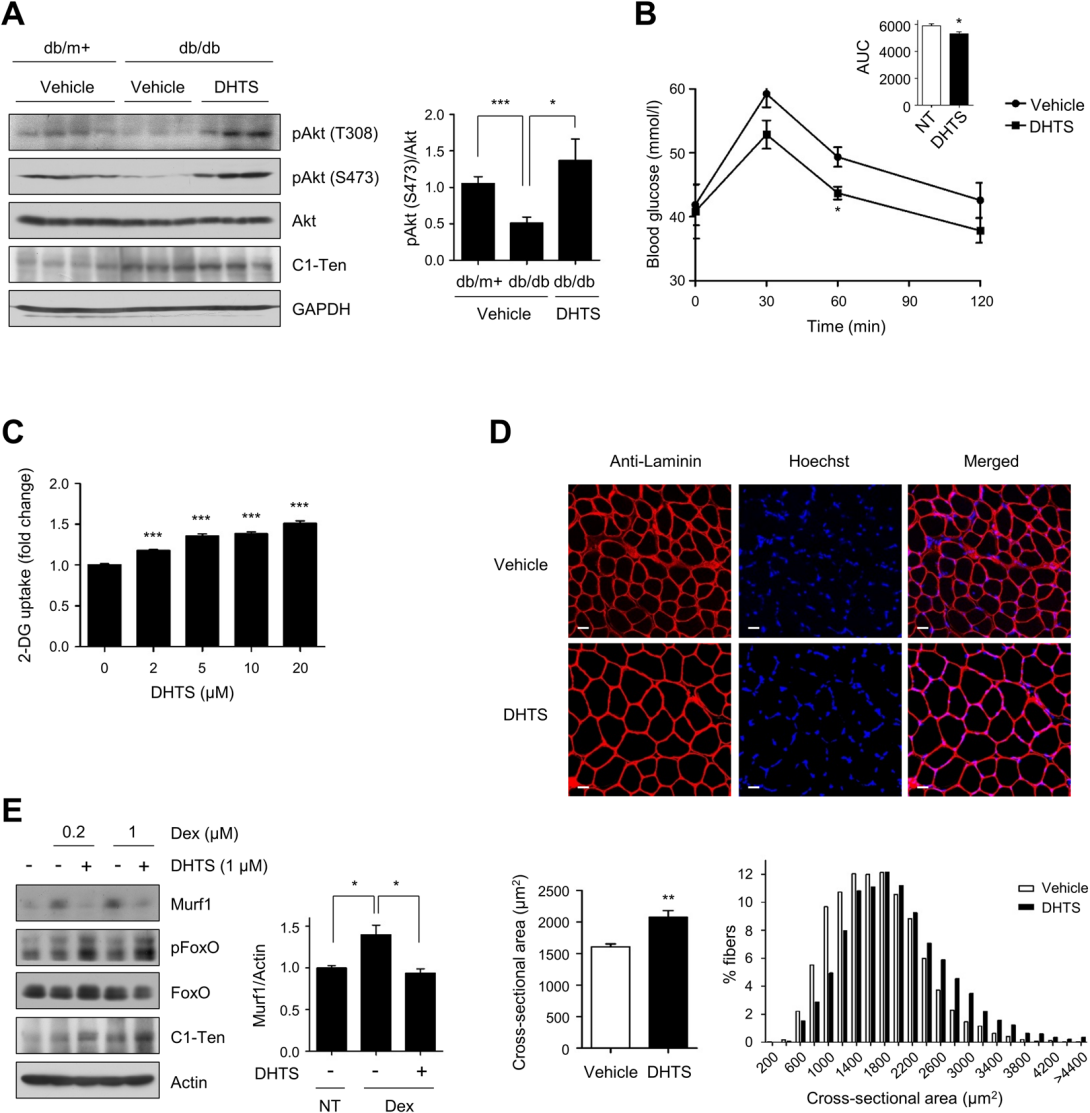
DHTS improves diabetic phenotypes. (**A**) Effects of DHTS on Akt phosphorylation in the skeletal muscle of db/db mice. (Right) Relative Akt phosphorylation (S473) normalized to total Akt. Data are presented as the mean ± SEM (n = 8–9); *P < 0.05 and ***P < 0.001. (**B**) Oral glucose tolerance test (OGTT) and area under the curve (AUC) in vehicle- and DHTS-treated db/db mice. Data are presented as the mean ± SEM (n = 4); *P < 0.05. (**C**) Effects of DHTS on glucose uptake in myotubes. Dexamethasone-treated L6 myotubes were incubated with the indicated concentrations of DHTS for 1 h, and glucose uptake was measured. Data are presented as the mean ± SEM of six biological replicates, and similar results were obtained from three independent experiments; ***P < 0.001. (**D**) Effects of DHTS on muscle atrophy in db/db mice. Representative images of tibialis anterior (TA) muscle sections stained with antibody to laminin and Hoechst 33342. The images were taken at 25× magnification; scale bar = 20 μm. Mean cross-sectional area (CSA) of TA muscle fibers (bottom left). Frequency distribution of the CSA of fibers plotted as frequency histograms (bottom right). More than 1000 myofibers from two or three sections per mouse were measured. Data are presented as the mean ± SEM (n = 5); **P < 0.01. (**E**) DHTS-induced inhibition of Murf1 expression. After L6 myotubes were incubated with DMSO or 1 μM DHTS for 24 h, the cells were cotreated with dexamethasone for 24 h. Data are presented as the mean ± SEM (n = 4); *P < 0.05.

To validate the functional effect of DHTS on C1-Ten, we first examined whether DHTS could improve pathological phenotypes in type 2 diabetes. Insulin resistance impairs glucose tolerance and induces muscle atrophy in type 2 diabetes, and Akt activation is often impaired in insulin-resistant muscle^13,14^. Consistent with previous findings^3^, C1-Ten levels were higher in the skeletal muscle of db/db mice, and Akt phosphorylation levels were lower in these tissues than in those from control (db/m+) mice (Fig. 2A). Administration of DHTS in db/db mice restored Akt phosphorylation to the levels observed in control mice (Fig. 2A), and improved oral glucose tolerance (Fig. 2B). Akt activation is important for glucose disposal in skeletal muscle; dexamethasone has been shown to induce insulin resistance in skeletal muscle by upregulating C1-Ten and decreasing Akt activation (Supplementary Fig. S2A)^3^. To validate the role of DHTS in glucose use of muscle cells, glucose uptake was measured in L6 myotubes under dexamethasone-induced insulin-resistant conditions. DHTS increased glucose uptake in a dose- and time-dependent manner in differentiated L6 myotubes (Fig. 2C, Supplementary Fig. S2B). Furthermore, we used a homogeneous population of differentiated myocytes derived from primary prepared EDL myoblasts to investigate whether DHTS stimulates glucose uptake in the primary cells as in the cell lines. Incubation of primary myocytes from EDL muscles with DHTS stimulated glucose uptake (Supplementary Fig. S2C).

Next, we assessed the effects of DHTS on muscle atrophy in db/db mice. DHTS treatment caused a 30% increase in the mean cross-sectional area and a rightward shift in the distribution of myofiber cross-sectional area in db/db mice (Fig. 2D). Upregulated C1-Ten causes muscle atrophy by activating FoxO, thus inducing a high-fidelity marker of muscle atrophy, muscle-specific E3 ligase (muscle RING finger 1 [MuRF-1])^3^. We investigated whether DHTS could prevent MuRF-1 induction by inhibiting FoxO (inducing FoxO phosphorylation). The results show that DHTS induced FoxO phosphorylation and prevented MuRF-1 expression in L6 myotubes, despite the dexamethasone-induced increase in C1-Ten (Fig. 2E), suggesting that DHTS blocks the action of C1-Ten. Taken together, we demonstrated that DHTS improves glucose metabolism and muscle atrophy in the insulin-resistant state using *in vitro* and *in vivo* models.

### DHTS increases IRS-1 stability

**Figure 3:**
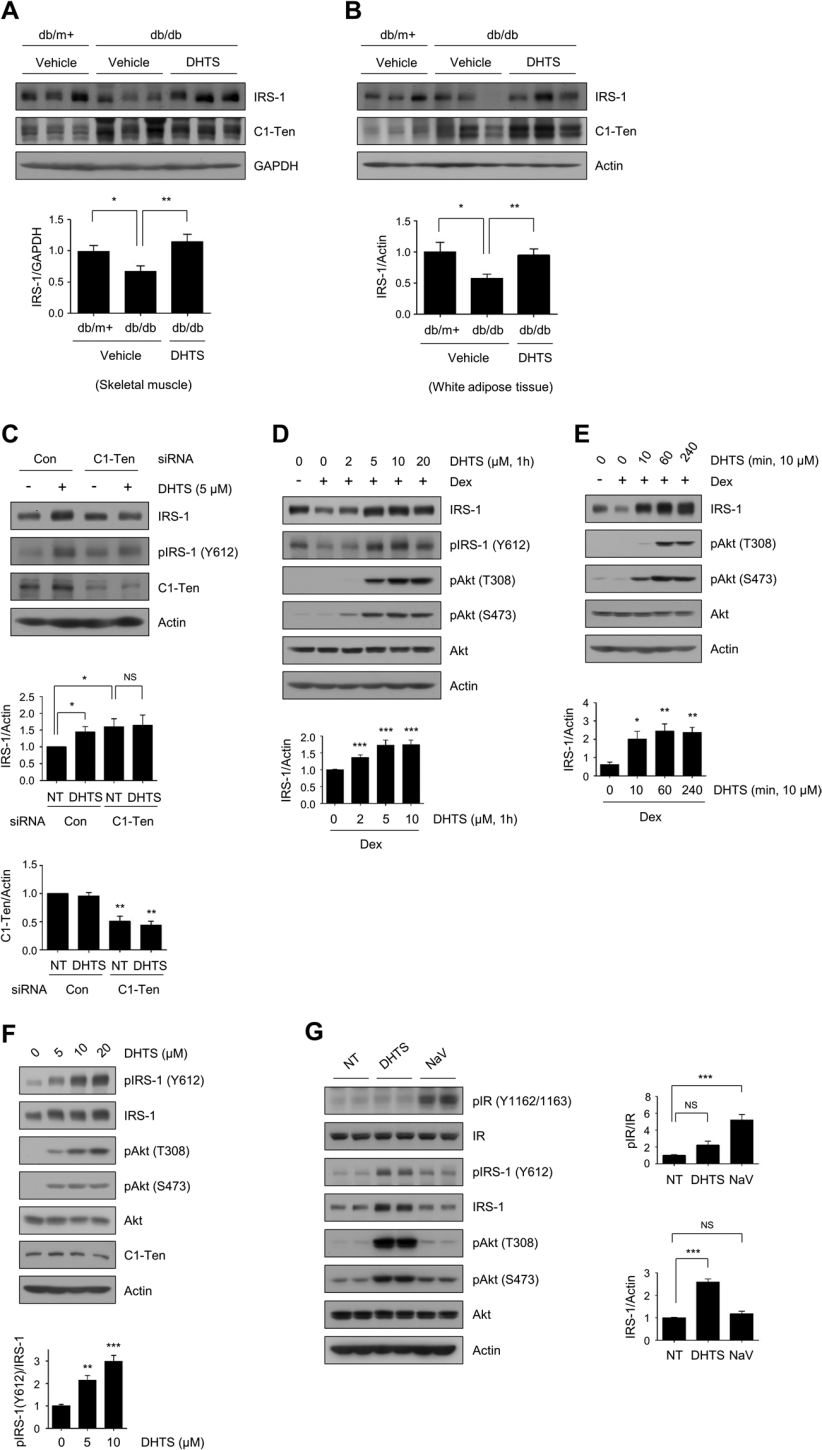
DHTS increases IRS-1 protein levels via C1-Ten inhibition. (**A**) IRS-1 protein levels in the skeletal muscle of db/db mice after treatment with vehicle or DHTS. Relative IRS-1 protein levels normalized by GAPDH (bottom). Data are presented as the mean ± SEM (n = 8–9); *P < 0.05 and **P < 0.01. (**B**) IRS-1 protein levels in the white adipose tissue of db/db mice after treatment with vehicle or DHTS. Relative IRS-1 protein levels normalized to actin expression (bottom). Data are presented as the mean ± SEM (n = 8–9); *P < 0.05 and **P < 0.01. (**C**) Effect of C1-Ten knockdown on DHTS-induced IRS-1 recovery in L6 myoblasts. L6 myoblasts were transfected with 100 nM siRNA targeting C1-Ten. At 48 h post-transfection, the cells were treated with 5 μM DHTS. Data are presented as the mean ± SEM (n = 4); *P < 0.05 and **P < 0.01; NS, not significant. (**D** and **E**) Effect of DHTS on IRS-1 protein levels in L6 myotubes. Fully differentiated L6 myotubes were incubated with 200 nM dexamethasone for 24 h. DHTS was added to the cells (**D**) for 1 h at the indicated concentrations, or (**E**) with 10 μM for the indicated time periods. Data are presented as the mean ± SEM (n = 7–8 and n = 4, respectively); *P < 0.05, **P < 0.01, and ***P < 0.001. (**F**) Effect of DHTS on IRS-1 Y612 phosphorylation in L6 myoblasts. Indicated concentrations of DHTS were administered to L6 myoblasts for 1 h at 48 h post-seeding. Data are presented as the mean ± SEM (n = 4); **P < 0.01 and ***P < 0.001. (**G**) Comparison of DHTS with NaV for the effects on insulin signaling. L6 myotubes were incubated with 200 nM dexamethasone for 24 h, followed by incubation with DMSO (NT), DHTS (10 μM), or NaV (1 mM) for 1 h. Data are presented as the mean ± SEM (n = 4); ***P < 0.001; NS, not significant.

C1-Ten reduces IRS-1 protein levels via its PTPase activity^3^. Therefore, a potential mechanism for the observed improvement in diabetic phenotypes in this study might be that DHTS increased IRS-1 levels. IRS-1 is a key protein that transduces insulin signals in skeletal muscle and white adipose tissue (WAT)^15^. We revealed that both of these tissues from db/db mice had relatively low IRS-1 protein levels but relatively high C1-Ten levels (Fig. 3A,B), supporting the notion that the mechanism of insulin resistance is controlled at the point of IRS-1 levels. DHTS treatment significantly restored IRS-1 levels without affecting C1-Ten levels in skeletal muscle (Fig. 3A) and WAT from db/db mice (Fig. 3B). To verify whether DHTS can regulate IRS-1 protein stability through C1-Ten inhibition, we treated L6 myoblasts and myotubes with DHTS after silencing C1-Ten. As *in vivo*, DHTS increased IRS-1 protein levels, phenocopying the C1-Ten knockdown effects; however, DHTS did not further increase IRS-1 levels in the absence of C1-Ten in myoblasts (Fig. 3C) and myotubes (Supplementary Fig. S3A). These results indicate that DHTS regulates IRS-1 levels via C1-Ten, supporting the role of DHTS in the direct inhibition of C1-Ten activity *in vitro*.

To characterize DHTS in IRS-1/Akt signaling further, we induced insulin resistance in L6 myotubes by exposing them to dexamethasone for 24 h. As reported previously^3,16^, dexamethasone reduced IRS-1 protein levels. However, this decrease in IRS-1 levels was blocked by DHTS incubation without altering *Irs-1* mRNA levels (Fig. 3D,E, Supplementary Fig. S3B), suggesting that DHTS affects IRS-1 protein stability. Increased IRS-1 protein levels were sustained up to 4 h (Fig. 3E). As IRS-1 is upstream of Akt, DHTS also enhanced Akt phosphorylation (Fig. 3D,E), consistent with DHTS-induced Akt activation in the skeletal muscle of db/db mice (Fig. 2A). Previously, we reported that C1-Ten-mediated IRS-1 Y612 dephosphorylation reduced IRS-1 stability. We used L6 myoblasts because myoblasts display a slower rate of IRS-1 turnover than myotubes, thereby providing the opportunity to monitor phosphorylation before its degradation occurred^3^. DHTS potentiated IRS-1 Y612 phosphorylation in a DHTS dose-dependent manner with increased Akt activation in L6 myoblasts (Fig. 3F). A general PTPase inhibitor, sodium orthovanadate (NaV), strongly potentiated insulin receptor tyrosine phosphorylation without increasing IRS-1 protein levels; however, DHTS exhibited stronger effects toward IRS-1 protein levels with almost no effect on insulin receptor tyrosine phosphorylation (Fig. 3G), implying that the DHTS-mediated increase in IRS-1 signaling was not mediated through general PTPase inhibition. These results demonstrate that DHTS restores IRS-1 stability and its downstream Akt signaling via inhibition of C1-Ten PTPase activity through a mechanism that differs from the action of a general PTPase inhibitor.

### DHTS targets both IRS-1 and AMPK pathways

**Figure 4:**
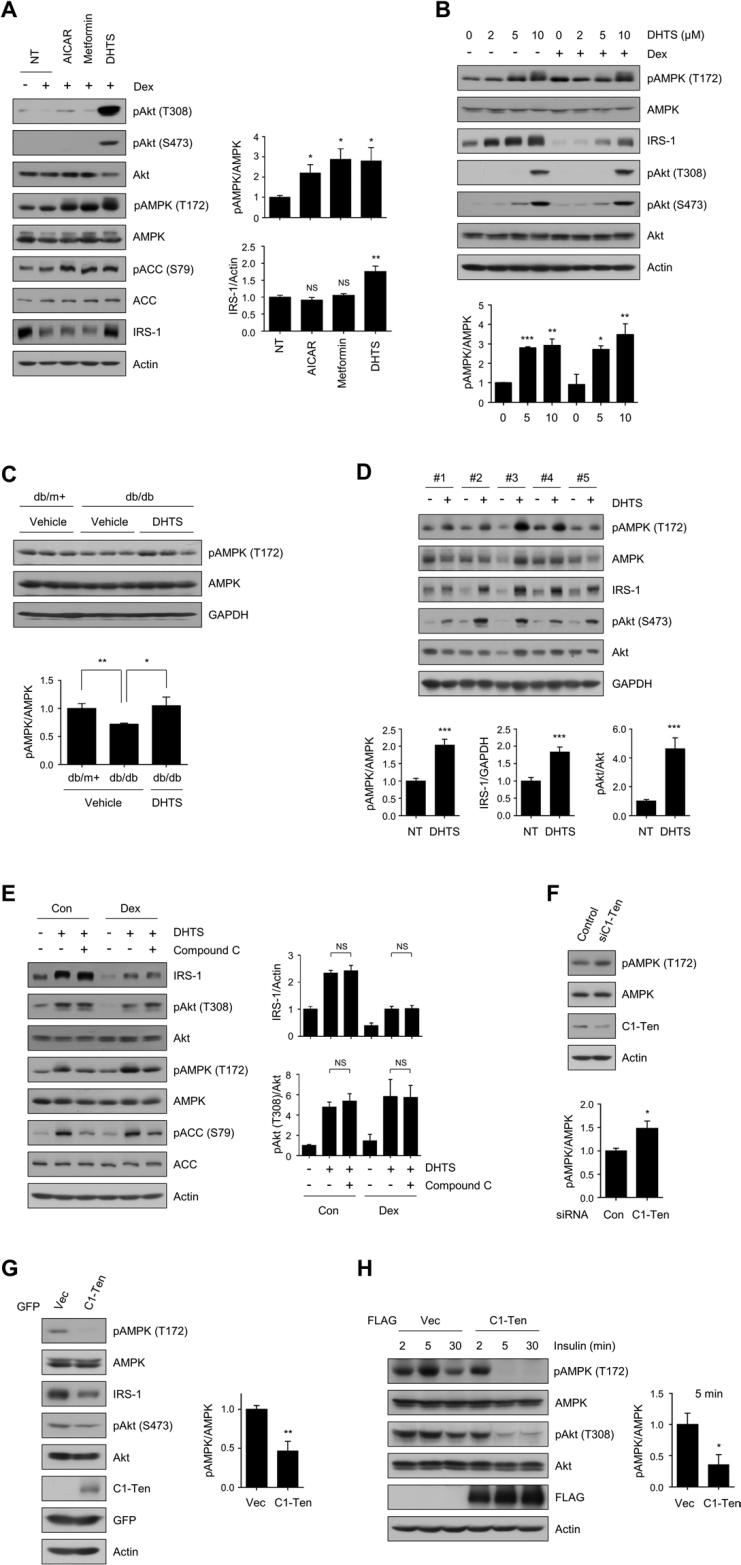
The C1-Ten inhibitor, DHTS, targets both IRS-1 and AMPK. (**A**) Effect of AMPK agonists on insulin signaling compared with that caused by DHTS. Fully differentiated L6 myotubes were incubated with 100 nM dexamethasone for 48 h followed by treatment with DMSO (NT), 5-aminoimidazole-4-carboxamide ribonucleotide (AICAR, 500 μM), metformin (5 mM), or DHTS (10 μM) for 4 h. Data are presented as the mean ± SEM (n = 4); *P < 0.05 and **P < 0.01; NS, not significant. (**B**) Changes in AMPK phosphorylation upon DHTS treatment in L6 myotubes. Fully differentiated L6 myotubes were incubated with 100 nM dexamethasone for 48 h followed by treatment with DHTS for 4 h. Data are presented as the mean ± SEM (n = 4–5); *P < 0.05, **P < 0.01 and ***P < 0.001. (**C**) Effect of DHTS on AMPK phosphorylation in the skeletal muscle of db/db mice. Relative AMPK phosphorylation (T172) normalized to total AMPK (bottom). Data are presented as the mean ± SEM (n = 8–9); *P < 0.05 and **P < 0.01. (**D**) EDL muscles from 9-week-old C57BL/6 J mice were incubated with medium containing 2 mM pyruvate in the presence of vehicle (DMSO) or 20 μM DHTS for 2 h. Data are presented as the mean ± SEM (n = 10); ***P < 0.001. (**E**) L6 myotubes were incubated with ethanol (Con) or 200 nM dexamethasone (Dex) for 24 h. After pretreatment with 10 μM Compound C for 30 min, the cells were cotreated with Compound C and DHTS (5 μM) for 10 min. Data are presented as the mean ± SEM (n = 4); NS, not significant. (**F**) Effect of C1-Ten knockdown on AMPK activation in L6 myotubes. L6 myotubes on day 3 were transfected with 100 nM control or C1-Ten siRNA. Data are presented as the mean ± SEM (n = 4); *P < 0.05. (**G**) Effect of C1-Ten on AMPK activation. L6 myotubes on day 7 were infected with adenovirus (Ad)-GFP or Ad-C1Ten for 48 h. Data are presented as the mean ± SEM (n = 4); **P < 0.01. (**H**) Effect of C1-Ten on AMPK signaling in HEK293 cells treated with insulin. HEK293 cells were transfected with FLAG-C1-Ten WT. After 24 h, the cells were serum starved for 18 h prior to treatment with 10 nM insulin. Data are presented as the mean ± SEM (n = 4); *P < 0.05.

We verified the potential antidiabetic property of the C1-Ten inhibitor, DHTS. Metformin is the most widely used first-line antidiabetic drug, which prevents insulin resistance by targeting the AMPK pathway^17,18^. In addition, pharmacological activation of AMPK can potentiate insulin signaling via PI3K/Akt activation^19^. Therefore, we compared the effects on insulin signaling of AMPK agonists (metformin or 5-aminoimidazole-4-carboxamide ribonucleotide, AICAR) and DHTS. As expected, AICAR- or metformin-induced AMPK activation increased Akt phosphorylation, but to a lesser extent than did DHTS under dexamethasone-induced insulin-resistant conditions (Fig. 4A). Surprisingly, DHTS induced AMPK activation comparable to the levels achieved by AICAR or metformin (Fig. 4A). Next, we examined the involvement of AMPK upstream kinases such as liver kinase B1 (LKB1) or Ca^2+^/calmodulin-dependent protein kinase kinase (CaMKK) in DHTS-mediated AMPK activation. First, we used HeLa cells, which do not express LKB1 protein (Supplementary Fig. S4A). DHTS-induced AMPK phosphorylation was also observed in HeLa cells. Next, cotreatment with CaMKK inhibitor STO-609 did not block DHTS-induced AMPK phosphorylation (Supplementary Fig. S4B). These results suggest that DHTS-mediated AMPK activation occurs in an LKB1- and CaMKK- independent manner. Instead of well-known AMPK upstream kinases, a novel pathway might be involved in this process. DHTS indeed increased AMPK phosphorylation in L6 myotubes in a dose-dependent manner (Fig. 4B). Moreover, DHTS administration increased AMPK activation in the skeletal muscle of db/db mice *in vivo* (Fig. 4C), as well as in isolated skeletal muscle of wild-type mice *ex vivo* (Fig. 4D, Supplementary Fig. S4C). In contrast, the AMPK agonist itself did not increase IRS-1 levels (Fig. 4A). These data indicate that AMPK activation *per se* is not the cause of enhanced IRS-1 stability by DHTS. To verify this, we incubated L6 myotubes with DHTS in the presence of the AMPK inhibitor, compound C. Although compound C efficiently blocked DHTS-induced AMPK activation, it did not inhibit the DHTS-mediated increase in IRS-1 levels and Akt phosphorylation (Fig. 4E), suggesting that DHTS restored the IRS-1/Akt pathway in an AMPK-independent manner.

Since DHTS mediated the activation of AMPK, we used siRNA knockdown of C1-Ten to test whether C1-Ten could inhibit AMPK. We observed AMPK activation in L6 myotubes expressing C1-Ten siRNA (Fig. 4F). Conversely, C1-Ten overexpression downregulated AMPK phosphorylation (Fig. 4G). Additionally, C1-Ten overexpression in HEK293 cells led to inhibition of the AMPK pathway and insulin-induced Akt activation (Fig. 4H). These results suggest that DHTS sensitizes insulin signaling by increasing IRS-1 stability and activating AMPK through C1-Ten inhibition.

### IRS-1 Y612 regulates both Akt and AMPK pathways

**Figure 5:**
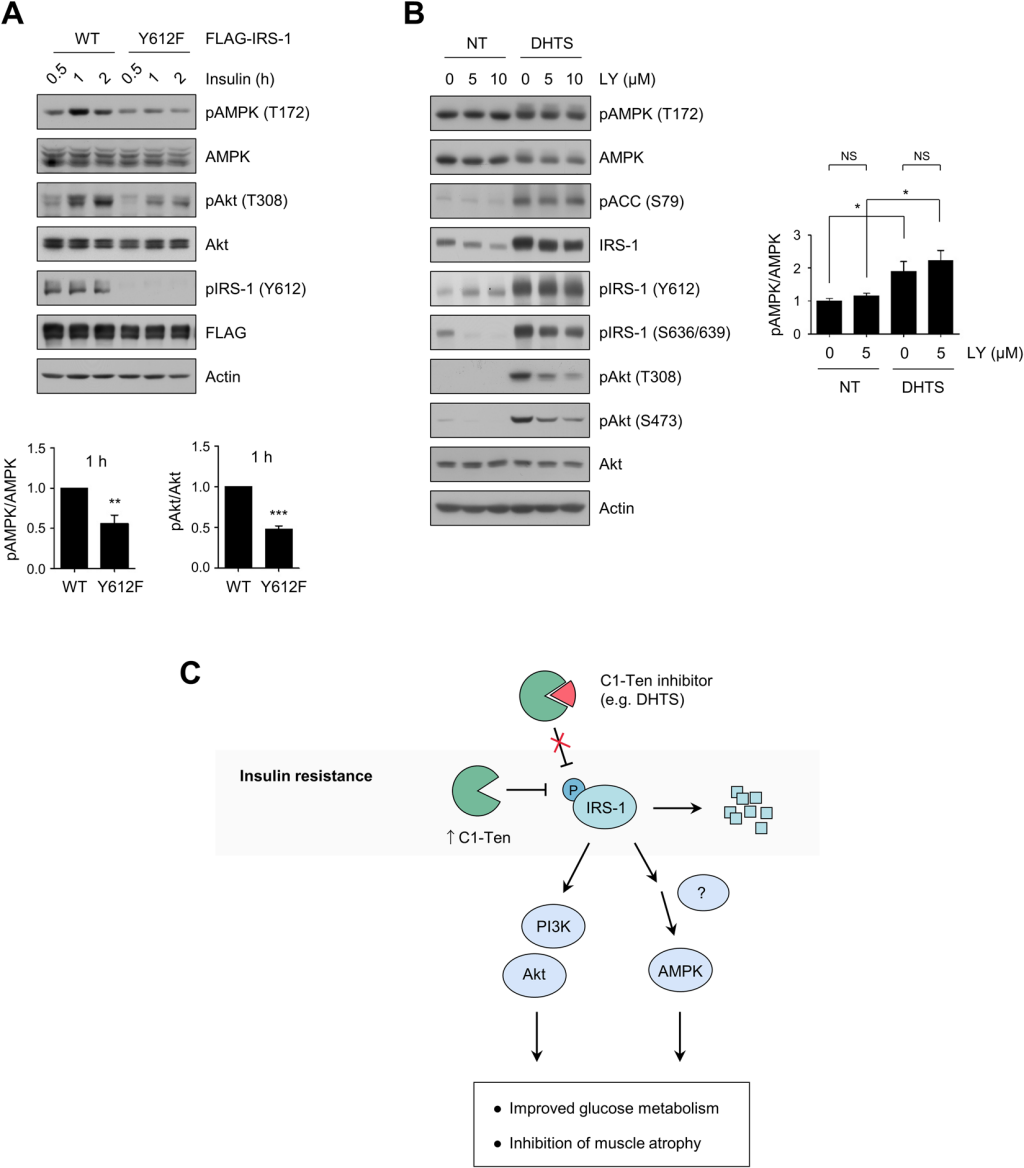
IRS-1 Y612 regulates both Akt and AMPK. (**A**) Effect of IRS-1 Y612F mutants on AMPK signaling. HEK293 cells were transfected with FLAG–IRS-1 WT or Y612F. The cells were serum starved for 18 h and stimulated with 10 nM insulin. Data are presented as the mean ± SEM (n = 5); **P < 0.01 and ***P < 0.001. (**B**) Role of PI3K on AMPK activation. Fully differentiated L6 myotubes were pretreated with LY294002 (LY) for 0.5 h. The cells were treated with DMSO (NT) or DHTS (10 μM) in the presence of LY. Data are presented as the mean ± SEM (n = 4); *P < 0.05; NS, not significant. (**C**) A proposed model: C1-Ten inhibition mediates IRS-1 and AMPK activation.

Knowing that DHTS-induced AMPK activation is not upstream of IRS-1/Akt signaling, we investigated whether IRS-1 Y612 phosphorylation could affect AMPK activation. Previously, we reported that the IRS-1 Y612F mutant mimics the effect of C1-Ten overexpression in HEK293 cells^3^. IRS-1 Y612F overexpression inhibited not only Akt activation but also AMPK activation (Fig. 5A), indicating that modulation of IRS-1 phosphorylation status itself is capable of regulating AMPK activity. IRS-1 Y612 is critical for PI3K activity^20^; thus, to determine whether PI3K activity is involved in modulating AMPK activation, myotubes were incubated with DHTS in the presence of the PI3K inhibitor, LY294002. LY294002 efficiently blocked DHTS-induced Akt activation without altering AMPK phosphorylation or IRS-1 protein levels (Fig. 5B), indicating that DHTS-induced AMPK activation is independent of DHTS-induced PI3K/Akt activation. Taken together, IRS-1 phosphorylation at the Y612 site is critical in both Akt activation and AMPK activation, emphasizing the roles of C1-Ten and its inhibitor, DHTS, in insulin resistance (Fig. 5C).

## Discussion

In this study, we identified DHTS as a C1-Ten PTPase inhibitor that improves glucose tolerance and muscle atrophy in db/db mice. DHTS increased IRS-1 stability, which activates Akt via C1-Ten inhibition. This identification of DHTS as a C1-Ten inhibitor revealed a novel role of C1-Ten in AMPK inhibition via IRS-1 Y612 dephosphorylation. Therefore, inhibition of C1-Ten-mediated IRS-1 degradation suggests a novel therapeutic strategy for insulin-resistance-associated metabolic syndrome via dual targeting of IRS-1/Akt and IRS-1/AMPK.

PTPases are highly vulnerable to oxidative and electrophilic inactivation due to the low pKa of their catalytic cysteines^12^. Reactive organic compounds, such as quinones, can generate ROS or directly impair PTPase activity either by thiolate oxidation or covalent modification of catalytic and regulatory residues. However, NAC treatment did not block DHTS-induced IRS-1 tyrosine phosphorylation (Fig. 1C), and DHTS increased IRS-1 tyrosine phosphorylation without ROS generation (Fig. 1D). Accordingly, the DHTS-induced C1-Ten inhibition was independent of ROS. Instead, we observed that DHTS inhibited the interaction between C1-Ten and IRS-1 *in vitro* (Supplementary Fig. S1C). In the case of PTP1B, 1,2-naphthoquinone (1,2-NQ) inactivates PTP1B through covalent attachment to the reactive cysteine in the active site^21^. DHTS may share a similar inhibition mechanism with 1,2-NQ since DHTS is a derivative of naphthoquinone. It is likely that covalent modification of DHTS would interrupt its accessibility to substrates. Although all of the active compounds identified in the inhibitor screening were naphthoquinone derivatives (Fig. 1A), the inhibitory activities of the compounds were different. In addition, DHTS has more propensity towards IRS-1, in contrast to general PTPase inhibitors, sodium orthovanadate, or PTP1B inhibitors with strong effects on insulin receptor phosphorylation (Fig. 3G).

According to the NCBI Gene Expression Omnibus (GEO) database, C1-Ten mRNA levels are upregulated in human skeletal muscle in insulin-resistant individuals (GDS ID: GDS3715), which is consistent with the results in diabetic mice^3^. However, in-depth analysis of C1-Ten expression levels in subjects with diabetes or other pathological conditions is still needed to understand the role of C1-Ten in those conditions. How C1-Ten expression is increased in the insulin-resistant condition remains elusive. A detailed mechanism for the upregulation of C1-Ten should be elucidated in further studies.

Insulin resistance in skeletal muscle plays a pivotal role in the pathogenesis of type 2 diabetes because muscle is the largest tissue of the human body, and 80% of whole-body insulin-stimulated glucose uptake occurs in muscle^22^. Impaired responsiveness to insulin typically occurs at the level of IRS-1, and this defect in IRS-1-mediated signaling has been observed in type 2 diabetes^1–5^. Therefore, maintenance of an appropriate IRS-1 level is essential for proper insulin signaling. A number of E3 ligases regulate IRS-1 stability and have been implicated in muscle atrophy and glucose intolerance^8,9,11,23^; however, inhibition of E3 ligase may result in unexpected side effects because of its non-canonical catalytic site and its broad-spectrum substrate specificity. To date, there are no known chemical inhibitors that regulate IRS-1 protein levels. Approaches other than E3 ligase targeting are necessary to prevent insulin resistance via IRS-1 regulation.

Along with IRS-1, AMPK is another attractive therapeutic target molecule. AMPK activation results in improved insulin sensitivity and maintenance of glucose homeostasis. In addition, accumulating evidence in mice and humans has highlighted the role of AMPK activation against diabetes^24,25^. Although AMPK activity and protein expression levels are generally normal in type 2 diabetes^26,27^, pharmacological activation of AMPK improves insulin sensitivity by stimulating glucose uptake and lowering blood glucose levels. Two classes of antidiabetic drugs, the biguanides (i.e., metformin) and the thiazolidinediones (i.e., rosiglitazone), improve insulin action by activating AMPK^17,28^. In addition, recent papers have shown that AMPK activation in skeletal muscle is a potent therapeutic approach for the treatment of diabetes. It was shown that new compounds (PF-739 and MK-8722) lower blood glucose levels through skeletal muscle AMPK activation in rodents and in non-human primates^29,30^. Therefore, the modulation of these two pathways (IRS-1 and AMPK) together may be an effective way to improve insulin sensitivity.

Although AMPK activation and increasing insulin sensitivity seem to be effective therapeutic approaches for insulin resistance, AMPK activation is the representative catabolic pathway and insulin is the representative anabolic hormone. However, many studies have suggested a close interconnection between the two pathways. AMPK sensitizes insulin signaling^19,31^ via the attenuation of mTORC1-mediated negative feedback towards IRS-1^32,33^ or by positively regulating PI3K/Akt^34^. A recent modeling study raised the possibility that the anabolic component, IRS, positively regulates the catabolic regulator, AMPK^35^, but no mechanism has been elucidated. Our study is the first to propose that IRS-1, and more specifically IRS-1 Y612 phosphorylation, might act upstream of AMPK. How IRS-1 Y612 phosphorylation regulates AMPK activation still remains to be elucidated. However, our findings provide proof-of-concept for targeting C1-Ten with DHTS, a 1,2-naphthoquinone derivative, as a potential therapeutic strategy for insulin resistance resulting from an imbalance between anabolism and catabolism.

## Methods

### Reagents and antibodies

All chemicals were purchased from Sigma-Aldrich (St. Louis, MO, USA) unless stated otherwise. The natural product library was acquired from Enzo Life Sciences (Farmingdale, NY, USA). Anti-Tenc1 antibody was obtained from GeneTex Inc. (Irvine, CA, USA) and Sigma-Aldrich. Anti-phospho-Akt (T308, S473), anti-phospho-IR (Y1150/1151), anti-phospho-FoxO1/3a (T24/T32), anti-FoxO1, anti-phospho-AMPKα (T172), AMPKα, and anti-ACC antibodies were purchased from Cell Signaling Technology (Danvers, MA, USA). Anti-phospho-IRS-1 (Y612), anti-IRS-1, and anti-phospho-ACC (S79) antibodies were obtained from Millipore (Darmstadt, Germany). Anti-Akt1/2, anti-Murf1, and anti-IR antibodies were purchased from Santa Cruz Biotechnology (Santa Cruz, CA, USA). 15,16-Dihydrotanshinone I (DHTS) for animal experiments was extracted and isolated from danshen as described below.

### Extraction and isolation of DHTS

Dried roots of *Salvia miltiorrhiza* (30.0 kg) were extracted with MeOH (25 L, 3×) at room temperature. After evaporation of the solvent under reduced pressure, crude MeOH extract (4.8 kg) was obtained and suspended in H_2_O, then partitioned with CHCl_3_ to obtain a CHCl_3_-soluble fraction (500.0 g). The CHCl_3_-soluble fraction was subjected to silica gel chromatography, eluted with a gradient mixture of *n*-hexane-CHCl_3_ from 20–100% CHCl_3_, and eight fractions were collected (Fr.1–Fr.8). Fr.4 (70.0 g) was subjected to an open column silica gel, eluted with methylene chloride, and eight fractions (Fr.4.1–Fr.4.8) were collected. Fr4.4 (20.0 g) was subjected to silica gel chromatography using *n*-hexane-EtOAc (10:1 → 1:1) to obtain four subfractions (Fr.4.4.1–Fr.4.4.4). Subfraction 4.4.2 (16.5 g) was further purified by silica gel chromatography using *n*-hexane-EtOAc (20:1 → 1:1) to produce DHTS (1.9 g).

### Incubation of isolated muscle tissue

Male C57BL/6J mice (5–9 weeks old) were kept on a 12 h light/dark cycle with free access to food and water. The mice were fasted overnight prior to the experiment. The mice were sacrificed by cervical dislocation, and soleus or EDL muscles were rapidly and carefully removed. Isolated muscles were incubated in α-minimal essential medium (α-MEM; Welgene, Daegu, South Korea) containing DHTS or vehicle (DMSO) for 2 h at 37 °C on a shaker. The muscles were frozen in liquid nitrogen immediately prior to sample preparation. All animal experiments were approved by the Pohang University of Science and Technology Institutional Animal Care and Use Committee (POSTECH IACUC) (Approval no. 2015-0037). All experiments were performed in accordance with the guidelines and regulations of the IACUC.

### Plasmid constructs

FLAG-C1-Ten WT, FLAG-IRS-1 WT, and FLAG-IRS-1 Y612F were generated as described previously^3^. Full length C1-Ten cDNA was subcloned into the *Not*I/*Eco*RV site of p3xFLAG-CMV10 using the following primers: 5′ -TTGCGGCCGCAATGAAGTCCAGC-3′ and 5′ -GCGATATCTCATCATTTTCTCTGGCCCAGTAG-3′.

### Recombinant Adenovirus

Adenovirus expressing C1-Ten WT was generated through homologous recombination between a linearized transfer vector pAd-Track-CMV vector carrying Flag–C1-Ten WT and the adenoviral backbone vector pAd-Easy as described previously^3^. The virus encoded the green fluorescent protein (GFP) transcribed from a second independent CMV promoter to monitor viral infection efficiency. Adenovirus coding for GFP alone (pAd-GFP) was used as a control. L6 myotubes were infected with adenoviruses for 48 h.

### Cell culture

FreeStyle 293-F cells (Invitrogen, Carlsbad, CA, USA) were cultured in FreeStyle 293 expression medium (Invitrogen) in accordance with the manufacturer’s instructions. To generate stable cell lines, the cells were transfected with 3× FLAG-tagged-C1-Ten WT construct using jetPEI reagent (Polyplus-transfection, Illkirch, France). Stable transfectants were selected with 100 μg/mL G418 (Geneticin; Gibco, Grand Island, NY, USA). HEK293 cells were maintained in Dulbecco’s modified Eagle’s medium (DMEM; Lonza, Basel, Switzerland) containing 10% fetal bovine serum (FBS; Lonza). L6 myoblasts were maintained in α-minimal essential medium (α-MEM; Welgene) supplemented with 10% FBS. Myoblasts were differentiated into myotubes in α-MEM supplemented with 2% FBS. After differentiation was induced, the medium was changed every other day until differentiation was completed.

### Transfection

Transfection was performed using Lipofectamine reagent (Invitrogen) or PEI 25000 (Polysciences, Warrington, PA, USA) according to the manufacturer’s protocol. For knockdown experiments, negative control small interfering RNA (siRNA) and C1-Ten siRNA (targeting the C1-Ten sequence TCAGTGGATTACAACATGACA) were used as described previously^3^. Cells were transfected with siRNA (100 nM) using Lipofectamine RNAiMAX reagent (Invitrogen) according to the manufacturer’s instructions.

### Western blotting

Frozen tissues and harvested cells were lysed in buffer containing 50 mM Tris-HCl (pH 7.4), 150 mM NaCl, 1 mM EDTA, 1 mM Na_3_VO_4_, 20 mM NaF, 10 mM glycerophosphate, 1 mM PMSF, 10% glycerol, 1% Triton X-100, 0.2% sodium deoxycholate, and protease inhibitor cocktail. Tissue samples were homogenized using the Tissue Lyser II (Qiagen, Hilden, Germany). The lysates were centrifuged at 14,000 rpm for 15 min. The resultant supernatants were subjected to SDS-PAGE on 6–16% gradient gels, followed by Western blot analysis. In brief, the proteins were transferred to nitrocellulose membranes (GE Healthcare, Buckinghamshire, UK). After blocking with 5% skim milk in TTBS buffer, the membranes were incubated with primary antibodies overnight at 4 °C. The membranes were then incubated with horseradish peroxidase-conjugated secondary antibodies at room temperature for 1 h. Signals were detected by enhanced chemiluminescence (ECL; Thermo Fisher Scientific, Waltham, MA, USA).

### Quantitative real-time PCR analysis

Total RNA was isolated from L6 myotubes using TRIzol reagent (iNtRON Biotechnology, Sungnam, South Korea), and 3 μg of RNA was reverse transcribed to cDNA with M-MLV reverse transcriptase (Promega, Madison, WI, USA). Quantitative PCR analysis was performed using SYBR Green I Nucleic Acid Gel Stain (Invitrogen) and a C1000 thermal cycler (Bio-Rad Laboratories, Hercules, CA, USA). The PCR was carried out in a final volume of 20 μL. The relative quantity of mRNA was calculated using the comparative Ct method after normalization to GAPDH. The sequences of the primers used in PCR are as follows:

IRS-1: 5′-GTGAACCTAAGTCCCAACCATAAC-3′ and 5′-CCGGCAGCCTTGAGTGTCT-3′

C1-Ten: 5′-CTCGGTGGAGTTTGTCTTCTCCTC-3′ and 5′-GCTGGTTGAAGTTTTCATAGGAGTC-3′

GAPDH: 5′-CCATGACAACTTTGGCATCG-3′ and 5′-CCTGCTTCACCACCTTCTTG-3′

### Glucose uptake

Glucose uptake assays were performed as described previously^36^. Briefly, differentiated L6 myotubes were treated with DHTS for the indicated times or concentrations. After washing twice with Krebs buffer, the cells were incubated with 2-deoxy[^14^C] glucose for 10 min. The reaction was terminated by washing three times with ice-cold phosphate-buffered saline (PBS) containing 25 mM D-glucose. The cells were lysed using a solution of 0.1 N NaOH and 0.1% SDS. Glucose levels were measured by liquid scintillation counter.

### *Ex vivo* glucose uptake

Primary EDL muscles were obtained from the forelimbs of 3- to 4-week-old littermate pups as described previously^37^. Dissected and minced muscle was enzymatically disaggregated in PBS containing 1.5 U/mL dispase II and 1.4 U/mL collagenase D (Roche, Penzberg, Germany) at 37 °C, and triturated with a 10-mL pipette every 5 min for 20 min. Cells were filtered through 70-µm mesh (BD, Seoul, South Korea) and collected by pelleting at 1,000 rpm for 5 min. The cell pellet was dissociated in F10 medium (Invitrogen) supplemented with 10 ng/mL basic fibroblast growth factor (PeproTech, Rocky Hill, NJ, USA) and 10% fetal calf serum (Hyclone, Logan, UT, USA). Finally, cells were pre-plated twice onto non-collagen-coated plates for 1 h each to deplete the fibroblasts. After cells reached confluence, differentiation was induced by incubation in DMEM supplemented with 2% FBS for 2 days. Cells were washed twice with PBS and starved in serum-free low-glucose DMEM for 3 h. Cells were incubated with KRB (20 mM HEPES [pH 7.4], 130 mM NaCl, 1.4 mM KCl, 1 mM CaCl_2_, 1.2 mM MgSO_4_, and 1.2 mM KH_2_PO_4_), and incubated with the indicated compounds at 37 °C. The uptake assay was initiated by adding 2-deoxy-d(H^3^)-glucose (2-DG) to each well and incubating at 37 °C for 15 min. The reaction was terminated by washing with ice-cold PBS. The cells were lysed in 10% SDS and mixed with a scintillation cocktail to measure radioactivity. The experiment was approved by the Korea University Institutional Animal Care Use Committee (KOREA-2017-0087-C1) and was performed in accordance with the guidelines and regulations of the IACUC.

### *In vitro* PTPase assay

The PTPase assay was performed as described previously^3^, with minor modifications. C1-Ten protein was purified from a stable FresStyle293-F cell line expressing 3× FLAG tagged-C1-Ten using a FLAG immunoprecipitation system. The cells were lysed in lysis buffer (50 mM Tris-HCl [pH 7.4], 150 mM NaCl, 1 mM EDTA, 10% glycerol, 1 mM PMSF, 20 mM NaF, 0.025% mercaptoethanol, 1% Triton X-100, 0.2% sodium deoxycholate, and a protease inhibitor mixture). The soluble fractions of the cell lysates were isolated by centrifugation at 14,000 rpm for 15 min at 4 °C. The supernatant was subjected to immunoprecipitation with FLAG M2 Affinity Gel for 4 h at 4 °C. After incubation, the immunoprecipitates were washed three times with lysis buffer and once with wash buffer (50 mM Tris-HCl, 50 mM NaCl, 10% glycerol, 5 mM DTT). Bound proteins were eluted with elution buffer (50 mM Tris-HCl, 50 mM NaCl, 10% glycerol, 5 mM DTT) containing 100 µg/mL of 3× FLAG peptide. After purification, the amount and activity of purified C1-Ten protein were confirmed ahead of each experiment. A malachite green assay kit (Millipore, Milford, MA) was used to measure the PTPase activity. Enzyme reactions were performed in a final volume of 50 μL of reaction buffer (50 mM Tris-HCl [pH 7.4], 50 mM NaCl, 5 mM DTT, 2 mM MgCl_2_) in 96-well plates. Purified C1-Ten protein was pre-incubated with DHTS or the indicated chemicals at 25 °C for 30 min. Enzyme reactions were performed at 30 °C for 90 min. The reaction was terminated by adding malachite green detection solutions. Absorbance was measured at 620 nm.

### Detection of intracellular ROS

Cells were loaded with 5 μM CM-H_2_DCFDA (Invitrogen) for 30 min. The cells were co-treated with NAC and the indicated compound (DMSO, 10 μM DHTS, 1 mM H_2_O_2_, or 25 μM Menadione) for 1 h. After trypsinization, the fluorescence intensity was measured by flow cytometry using a BD LSR Fortessa. At least 10,000 cells were counted for each sample. Data were analyzed with FlowJo software v10 (Tree Star, San Carlos, CA, USA).

### Animal experiments

Male leptin receptor-deficient (db/db) and control (db/m+) mice with C57BLKS/J background were purchased from Central Lab Animal Inc. (Seoul, South Korea) and maintained on a 12-h light/dark cycle with free access to food and water. To test whether DHTS could improve pathological phenotypes in type 2 diabetes such as glucose intolerance and muscle atrophy, 10-week-old mice were administered DHTS (300 mg/kg/day) for 2 weeks by oral injection. DHTS was dissolved in a vehicle of 1% carboxymethylcellulose (CMC). For the glucose tolerance test, overnight-fasted mice were administered orally with 1.0 g/kg glucose. Blood glucose levels were measured at the indicated time points using Accu-Chek Performa (Roche, Mannheim, Germany). The skeletal muscle and WAT were frozen immediately in liquid nitrogen and stored at –80 °C after sacrifice. All animal experiments were approved by the Pohang University of Science and Technology Institutional Animal Care and Use Committee (POSTECH IACUC) (Approval no. 2015-0037). All experiments were performed in accordance with the guidelines and regulations of the IACUC.

### Immunofluorescence

For immunohistochemical staining, tibialis anterior muscles were embedded and frozen in frozen section compound (FSC 22; Leica, Wetzlar, Germany). Cross sections (10 μm thick) were cut using a cryostat microtome (CM1850, Leica) at −20 °C. The cryosections were stained for laminin, and nuclei were visualized with Hoechst 33342 stain (Sigma-Aldrich). The sections were fixed with 4% paraformaldehyde at room temperature for 10 min, rinsed with PBS, permeabilized with 0.2% Triton X-100 for 5 min, and blocked with 3% bovine serum albumin (BSA) in PBS for 1 h. The sections were incubated with laminin antibody (1:200; Sigma-Aldrich) overnight at 4 °C, incubated with anti-rat IgG antibody conjugated to Alexa 594 (1:500; Invitrogen) at room temperature for 1 h, rinsed with PBS, and incubated with Hoechst 33342 (1:50000) at room temperature for 10 min. Images were obtained by using a Leica SP5 confocal microscope. The muscle fiber cross-sectional area (CSA) was determined using ImageJ software.

### Statistical analysis

All data are presented as means ± standard error of the mean. Comparisons between two groups were made by unpaired two-tailed Student’s *t*-tests. P < 0.05 was deemed to indicate statistical significance. Statistical analysis was performed using GraphPad Prism version 5.03 for Windows (GraphPad Software Inc., La Jolla, CA, USA).

## Electronic supplementary material

Supplementary Information

## References

[1] Rondinone CM (1997). Insulin receptor substrate (IRS) 1 is reduced and IRS-2 is the main docking protein for phosphatidylinositol 3-kinase in adipocytes from subjects with non-insulin-dependent diabetes mellitus. Proceedings of the National Academy of Sciences of the United States of America.

[2] Kerouz NJ, Horsch D, Pons S, Kahn CR (1997). Differential regulation of insulin receptor substrates-1 and -2 (IRS-1 and IRS-2) and phosphatidylinositol 3-kinase isoforms in liver and muscle of the obese diabetic (ob/ob) mouse. The Journal of clinical investigation.

[3] Koh A (2013). C1-Ten is a protein tyrosine phosphatase of insulin receptor substrate 1 (IRS-1), regulating IRS-1 stability and muscle atrophy. Molecular and cellular biology.

[4] Krook A (2000). Characterization of signal transduction and glucose transport in skeletal muscle from type 2 diabetic patients. Diabetes.

[5] Bjornholm M, Kawano Y, Lehtihet M, Zierath JR (1997). Insulin receptor substrate-1 phosphorylation and phosphatidylinositol 3-kinase activity in skeletal muscle from NIDDM subjects after *in vivo* insulin stimulation. Diabetes.

[6] DeFronzo RA, Tripathy D (2009). Skeletal Muscle Insulin Resistance Is the Primary Defect in Type 2 Diabetes. Diabetes care.

[7] Wang X, Hu Z, Hu J, Du J, Mitch WE (2006). Insulin resistance accelerates muscle protein degradation: Activation of the ubiquitin-proteasome pathway by defects in muscle cell signaling. Endocrinology.

[8] Rui L, Yuan M, Frantz D, Shoelson S, White MF (2002). SOCS-1 and SOCS-3 block insulin signaling by ubiquitin-mediated degradation of IRS1 and IRS2. The Journal of biological chemistry.

[9] Yi JS (2013). MG53-induced IRS-1 ubiquitination negatively regulates skeletal myogenesis and insulin signalling. Nature communications.

[10] Song R (2013). Central role of E3 ubiquitin ligase MG53 in insulin resistance and metabolic disorders. Nature.

[11] Scheufele F (2014). Evidence for a regulatory role of Cullin-RING E3 ubiquitin ligase 7 in insulin signaling. Cellular signalling.

[12] Ostman A, Frijhoff J, Sandin A, Bohmer FD (2011). Regulation of protein tyrosine phosphatases by reversible oxidation. Journal of biochemistry.

[13] Shao J, Yamashita H, Qiao L, Friedman JE (2000). Decreased Akt kinase activity and insulin resistance in C57BL/KsJ-Leprdb/db mice. The Journal of endocrinology.

[14] Krook A, Roth RA, Jiang XJ, Zierath JR, Wallberg-Henriksson H (1998). Insulin-stimulated Akt kinase activity is reduced in skeletal muscle from NIDDM subjects. Diabetes.

[15] Thirone AC, Huang C, Klip A (2006). Tissue-specific roles of IRS proteins in insulin signaling and glucose transport. Trends in endocrinology and metabolism: TEM.

[16] Ewart HS, Somwar R, Klip A (1998). Dexamethasone stimulates the expression of GLUT1 and GLUT4 proteins via different signalling pathways in L6 skeletal muscle cells. FEBS letters.

[17] Zhou G (2001). Role of AMP-activated protein kinase in mechanism of metformin action. The Journal of clinical investigation.

[18] Inzucchi SE (2015). Management of hyperglycemia in type 2 diabetes, 2015: a patient-centered approach: update to a position statement of the American Diabetes Association and the European Association for the Study of Diabetes. Diabetes care.

[19] Jessen N (2003). Effects of AICAR and exercise on insulin-stimulated glucose uptake, signaling, and GLUT-4 content in rat muscles. Journal of applied physiology.

[20] Esposito DL, Li Y, Cama A, Quon MJ (2001). Tyr(612) and Tyr(632) in human insulin receptor substrate-1 are important for full activation of insulin-stimulated phosphatidylinositol 3-kinase activity and translocation of GLUT4 in adipose cells. Endocrinology.

[21] Iwamoto N (2007). Chemical knockdown of protein-tyrosine phosphatase 1B by 1,2-naphthoquinone through covalent modification causes persistent transactivation of epidermal growth factor receptor. The Journal of biological chemistry.

[22] DeFronzo RA, Gunnarsson R, Bjorkman O, Olsson M, Wahren J (1985). Effects of insulin on peripheral and splanchnic glucose metabolism in noninsulin-dependent (type II) diabetes mellitus. The Journal of clinical investigation.

[23] Nakao R (2009). Ubiquitin ligase Cbl-b is a negative regulator for insulin-like growth factor 1 signaling during muscle atrophy caused by unloading. Molecular and cellular biology.

[24] Viollet B (2009). AMPK: Lessons from transgenic and knockout animals. Frontiers in bioscience.

[25] Boon H (2008). Intravenous AICAR administration reduces hepatic glucose output and inhibits whole body lipolysis in type 2 diabetic patients. Diabetologia.

[26] Hojlund K (2004). AMPK activity and isoform protein expression are similar in muscle of obese subjects with and without type 2 diabetes. American journal of physiology. Endocrinology and metabolism.

[27] Musi N (2001). AMP-activated protein kinase (AMPK) is activated in muscle of subjects with type 2 diabetes during exercise. Diabetes.

[28] LeBrasseur NK (2006). Thiazolidinediones can rapidly activate AMP-activated protein kinase in mammalian tissues. American journal of physiology. Endocrinology and metabolism.

[29] Myers RW (2017). Systemic pan-AMPK activator MK-8722 improves glucose homeostasis but induces cardiac hypertrophy. Science.

[30] Cokorinos EC (2017). Activation of Skeletal Muscle AMPK Promotes Glucose Disposal and Glucose Lowering in Non-human Primates and Mice. Cell Metab.

[31] Ouchi N (2004). Adiponectin stimulates angiogenesis by promoting cross-talk between AMP-activated protein kinase and Akt signaling in endothelial cells. The Journal of biological chemistry.

[32] Inoki K, Zhu T, Guan KL (2003). TSC2 mediates cellular energy response to control cell growth and survival. Cell.

[33] Gwinn DM (2008). AMPK phosphorylation of raptor mediates a metabolic checkpoint. Molecular cell.

[34] Tao R (2010). AMPK exerts dual regulatory effects on the PI3K pathway. Journal of molecular signaling.

[35] Sonntag AG, Dalle Pezze P, Shanley DP, Thedieck K (2012). A modelling-experimental approach reveals insulin receptor substrate (IRS)-dependent regulation of adenosine monosphosphate-dependent kinase (AMPK) by insulin. The FEBS journal.

[36] Yamamoto N (2015). Measurement of Glucose Uptake in Cultured Cells. Current protocols in pharmacolog.

[37] Bois PR, Grosveld GC (2003). FKHR (FOXO1a) is required for myotube fusion of primary mouse myoblasts. The EMBO journal.

