# Supplementary material for: Inhibition of C1-Ten PTPase activity reduces insulin resistance through IRS-1 and AMPK pathways
Source: Sci Rep. 2017 Dec 19;7:17777. doi: 10.1038/s41598-017-18081-8 (PMC5736594; doi:10.1038/s41598-017-18081-8)
Supplement: Supplementary file 1 — Supplementary Information [file 41598_2017_18081_MOESM1_ESM.pdf]

Supplementary Information

Inhibition of C1-Ten PTPase activity reduces insulin resistance through IRS-1 and AMPK pathways

Heeyoon Jeong[1], Ara Koh[1], Jiyoun Lee[1], Dohyun Park[1], Jung Ok Lee[2], Mi Nam Lee[1], Kyung-Jin Jo[1], Huynh Nguyen Khanh Tran[3], Eui Kim[4], Byung-Sun Min[3], Hyeon Soo Kim[2], Per-Olof Berggren[4,5], Sung Ho Ryu[1,4,*]

[1]Department of Life Sciences, Pohang University of Science and Technology, Pohang, 37673, Republic of Korea.

[2]Department of Anatomy, Korea University College of Medicine, Seoul, 02841, Republic of Korea.

[3]College of Pharmacy, Drug Research and Development Center, Catholic University of Daegu, Gyeongbuk, 38430, Republic of Korea.

[4]Division of Integrative Biosciences and Biotechnology, Pohang University of Science and Technology, Pohang, 37673, Republic of Korea.

[5]The Rolf Luft Research Center for Diabetes and Endocrinology, Karolinska Institutet, Karolinska University Hospital, S-171 76 Stockholm, Sweden.

[*]Correspondence: sungho@postech.ac.kr

Supplementary Figure S1

A

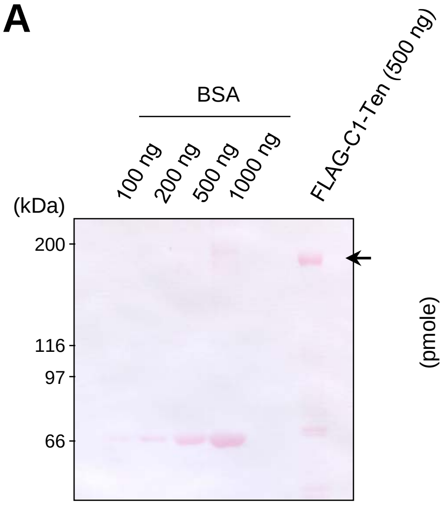

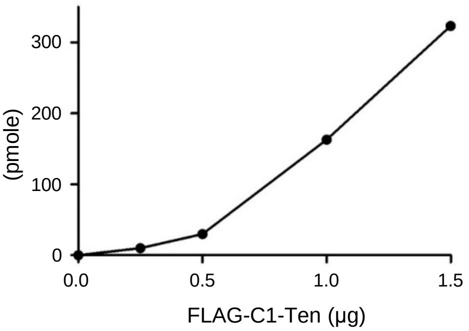

B

15,16-dihydrotanshinone I	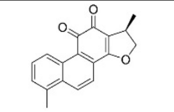
Tanshinone IIA	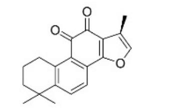
Cryptotanshinone	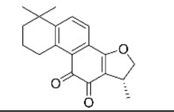
β-Lapachone	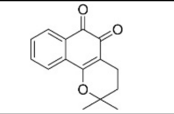
Shikonin	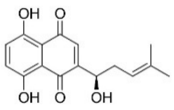
Plumbagin	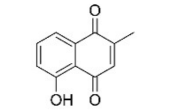
Streptonigrin	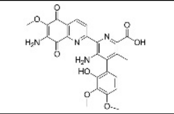
Menadione	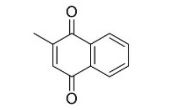
(β,β-Dimethylacryl) Shikonin	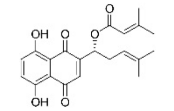
Deoxyshikonin	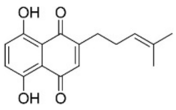

C

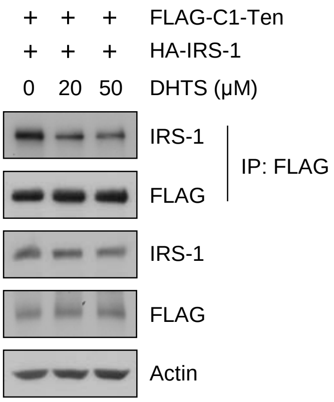

Supplementary Figure S2

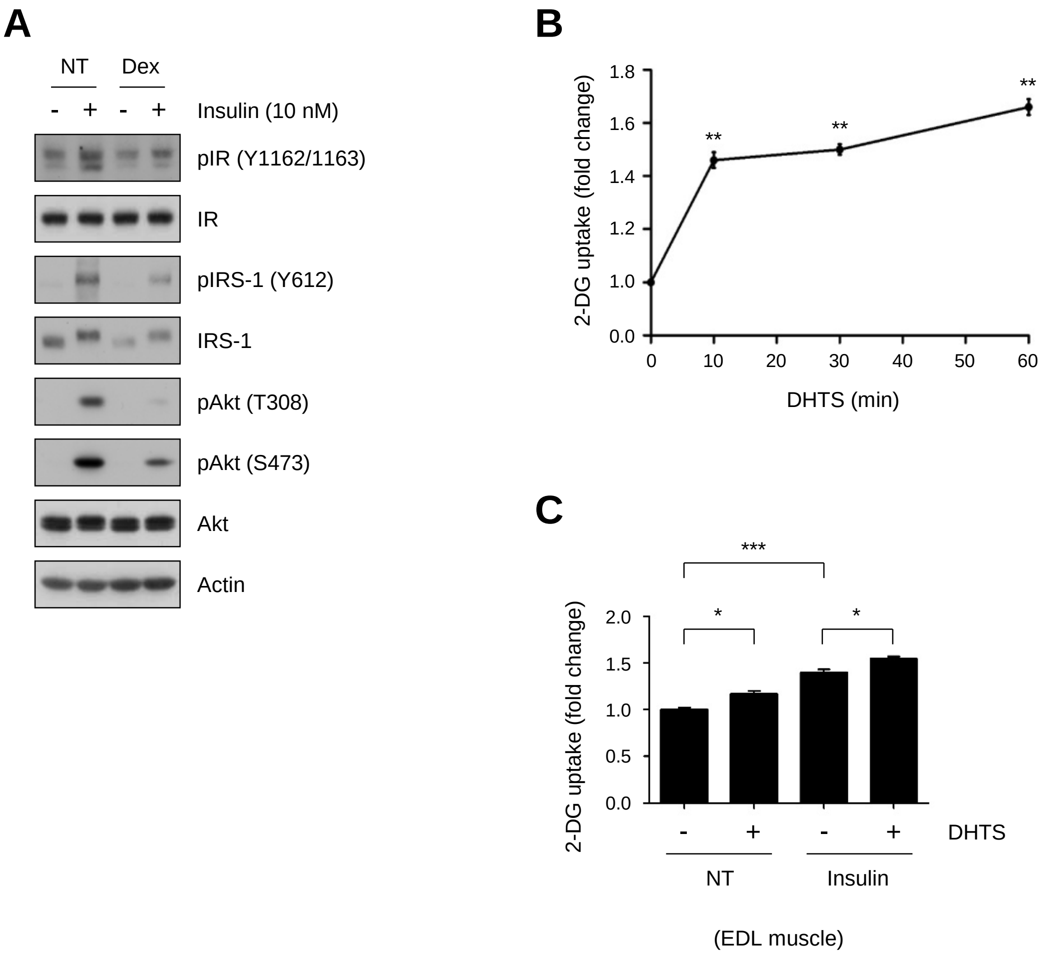

Supplementary Figure S3

A

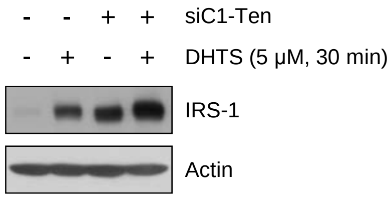

B

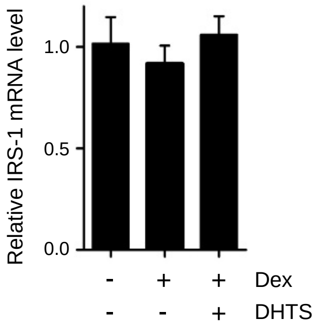

Supplementary Figure S4

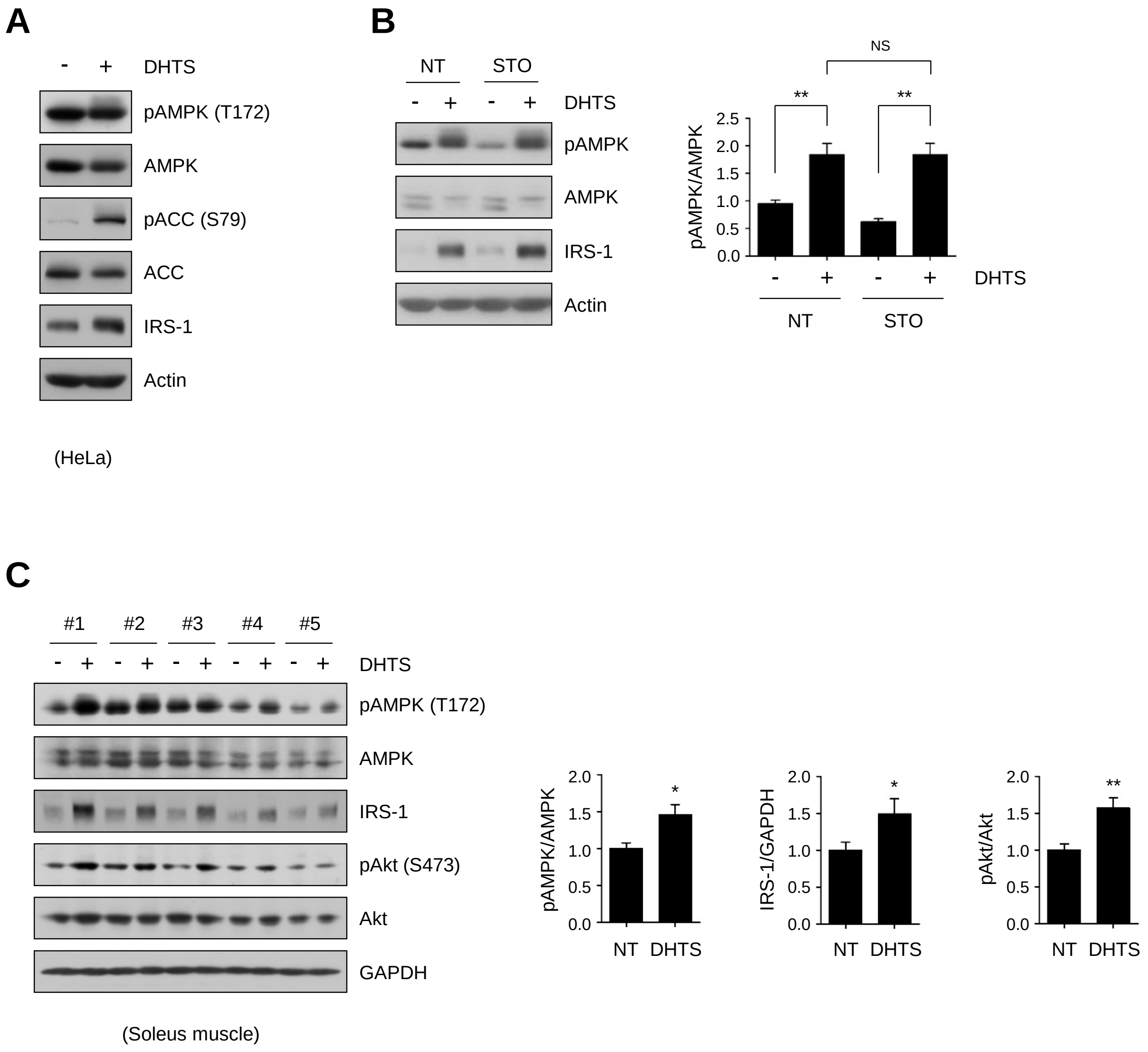

Supplementary Figure S5

Fig 1C

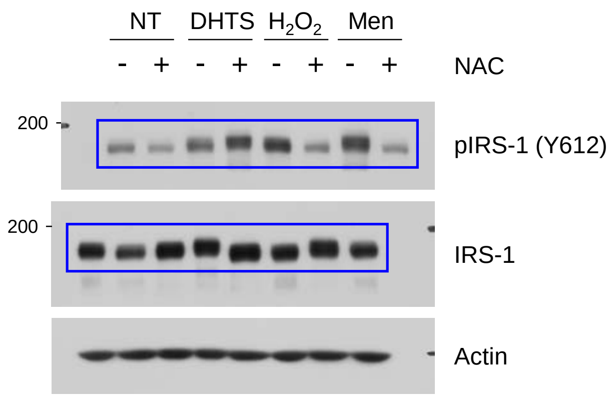

Supplementary Figure S5

Fig 2A

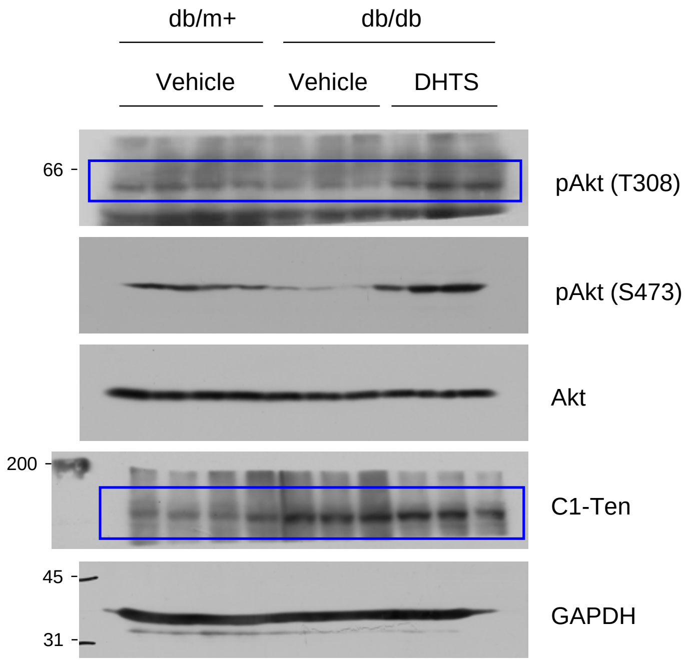

Fig 2E

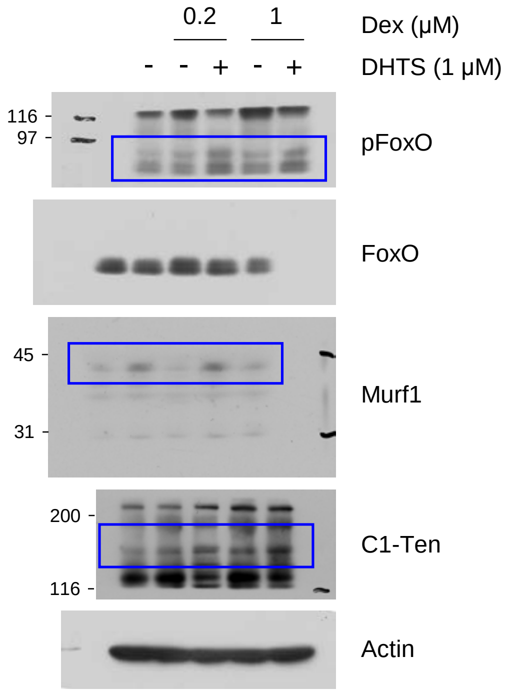

Fig 3A

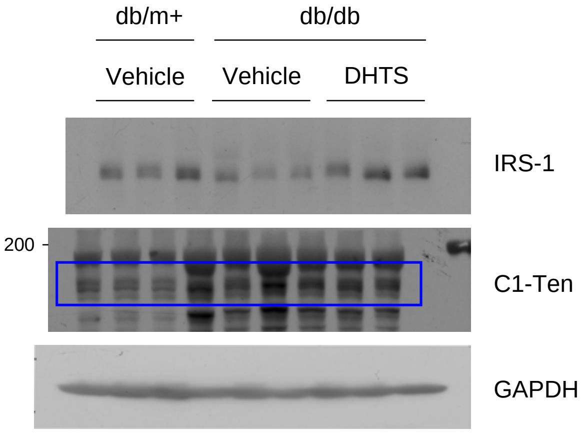

Fig 3B

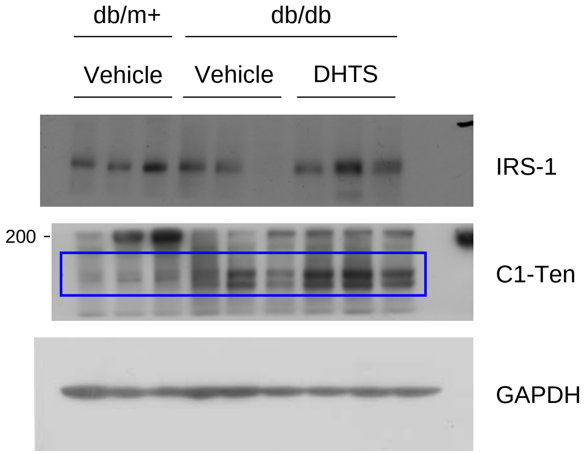

Supplementary Figure S5

Fig 3C

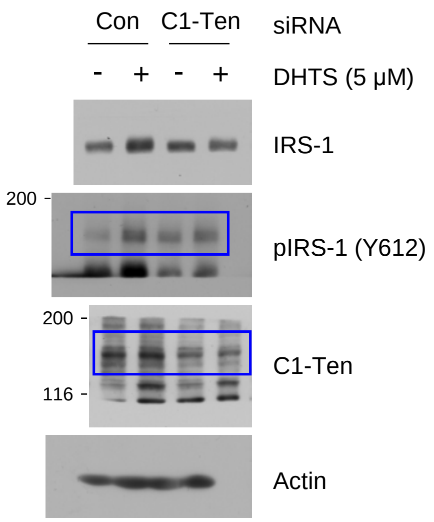

Fig 3D

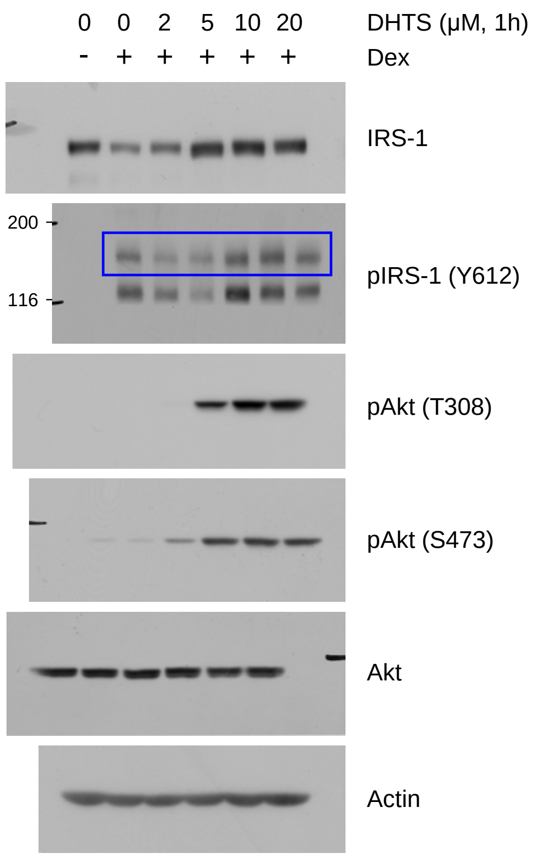

Fig 3E

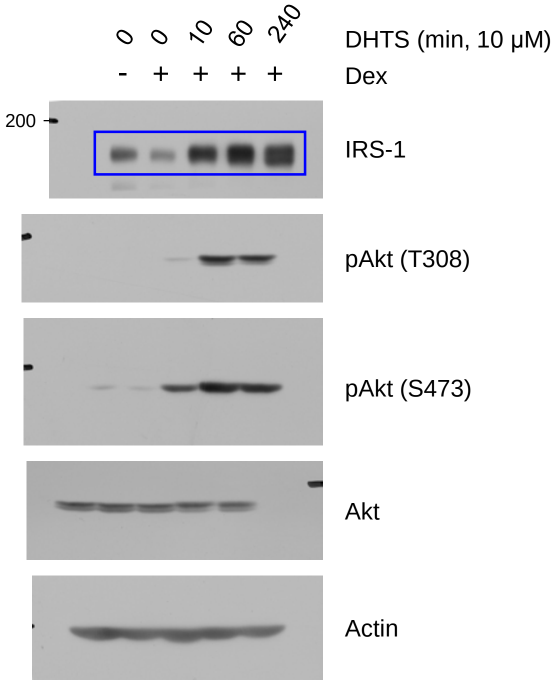

Supplementary Figure S5

Fig 3F

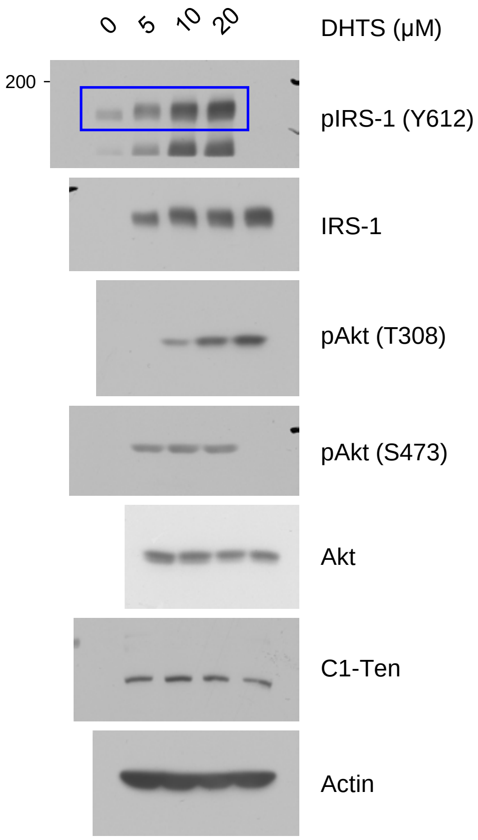

Fig 3G

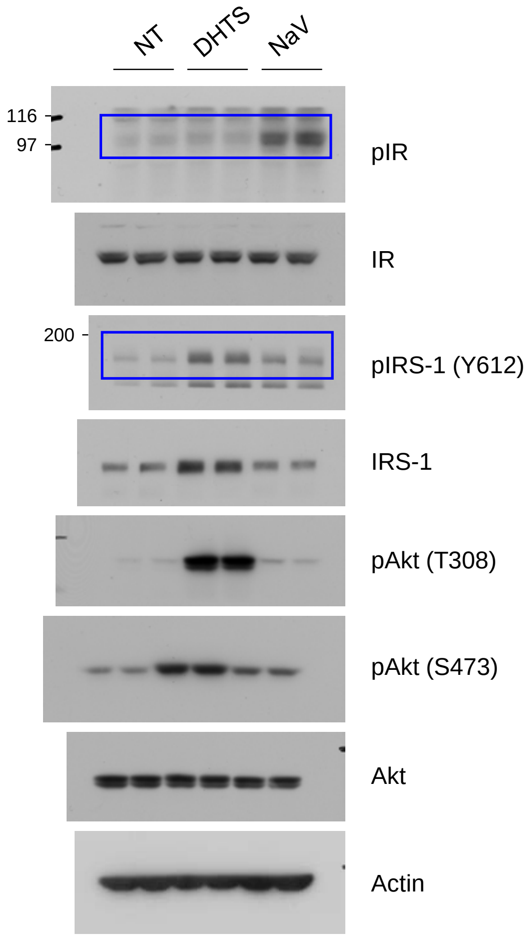

Supplementary Figure S5

Fig 4A

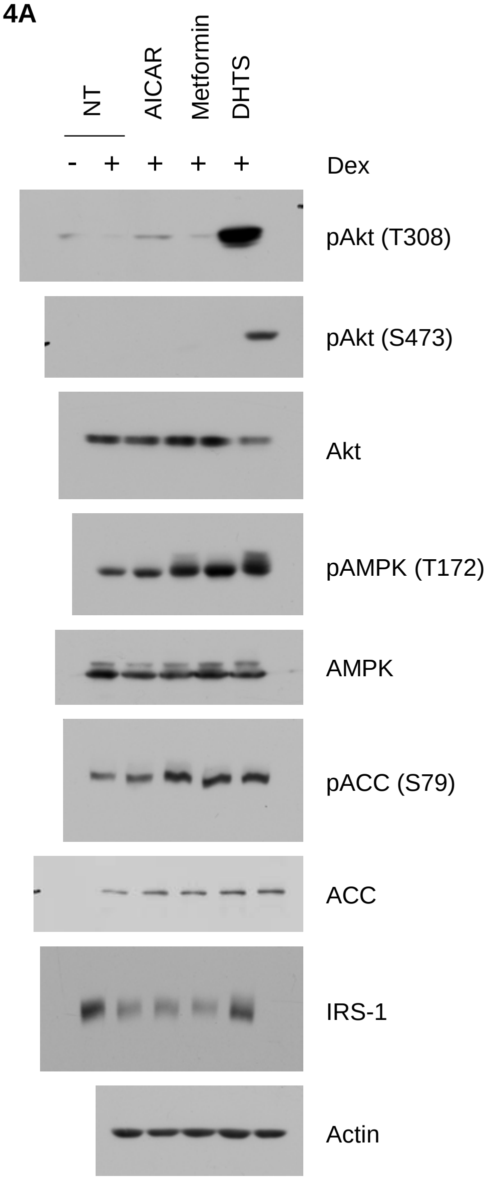

Fig 4B

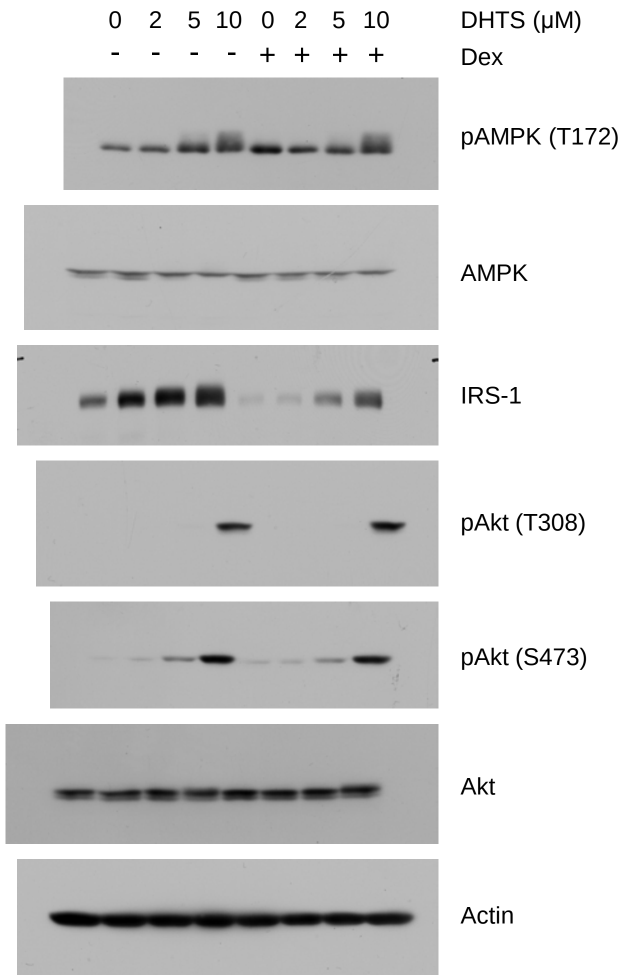

Fig 4C

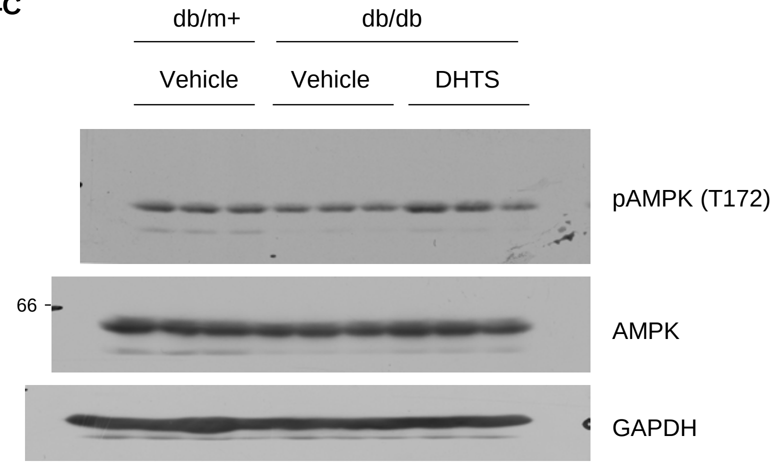

Supplementary Figure S5

Fig 4D

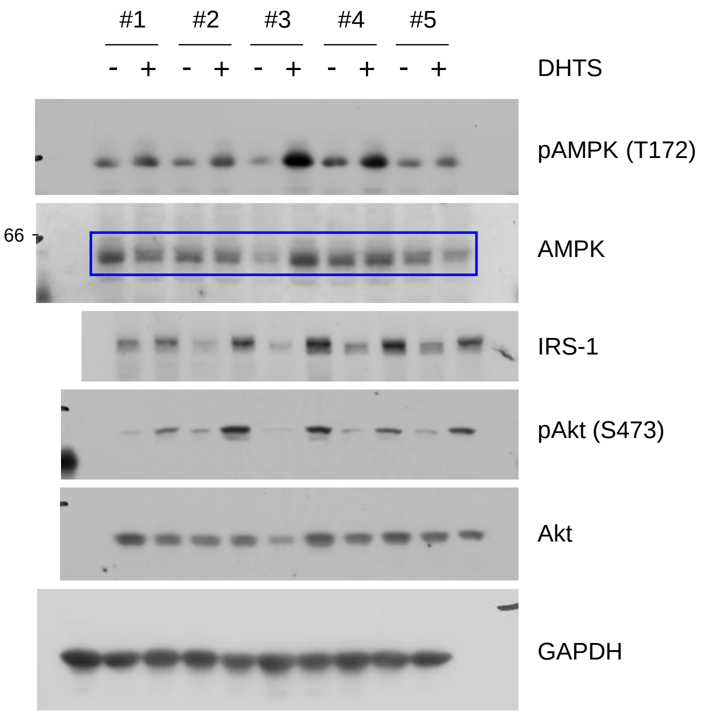

Fig 4E

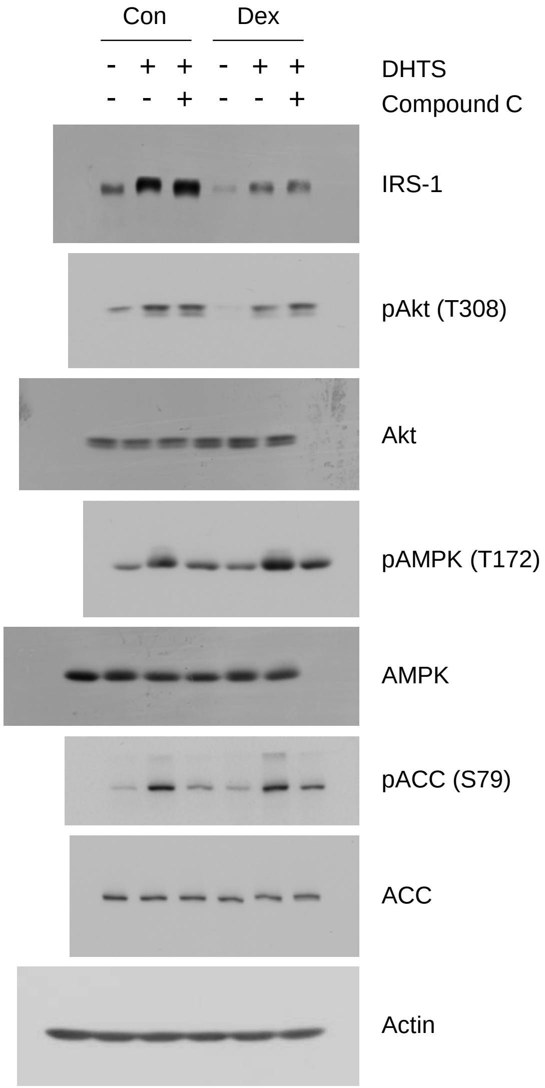

Supplementary Figure S5

Fig 4F

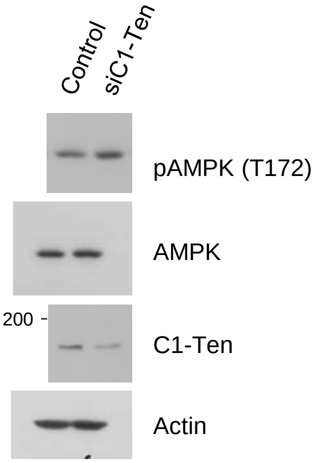

Fig 4G

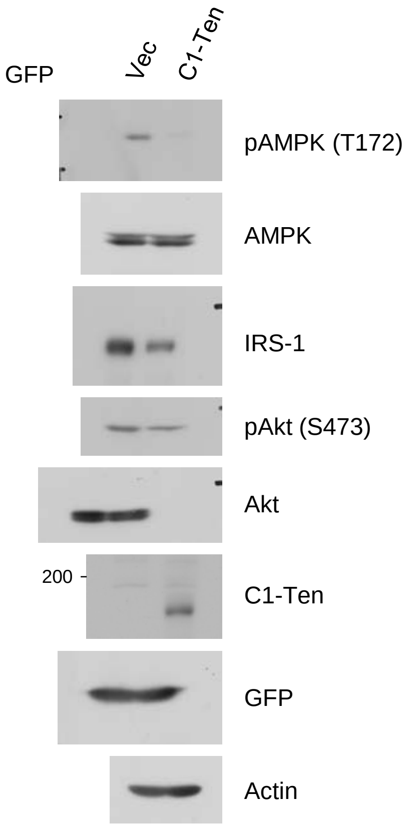

Fig 4H

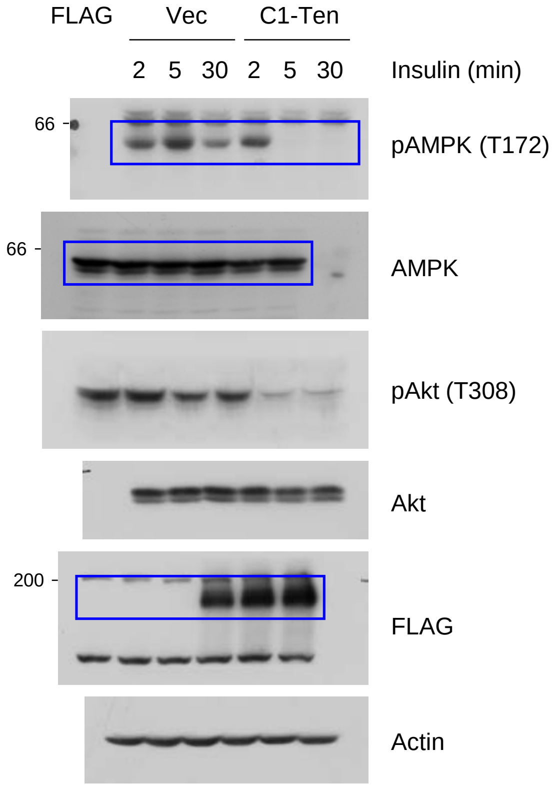

Supplementary Figure S5

Fig 5A

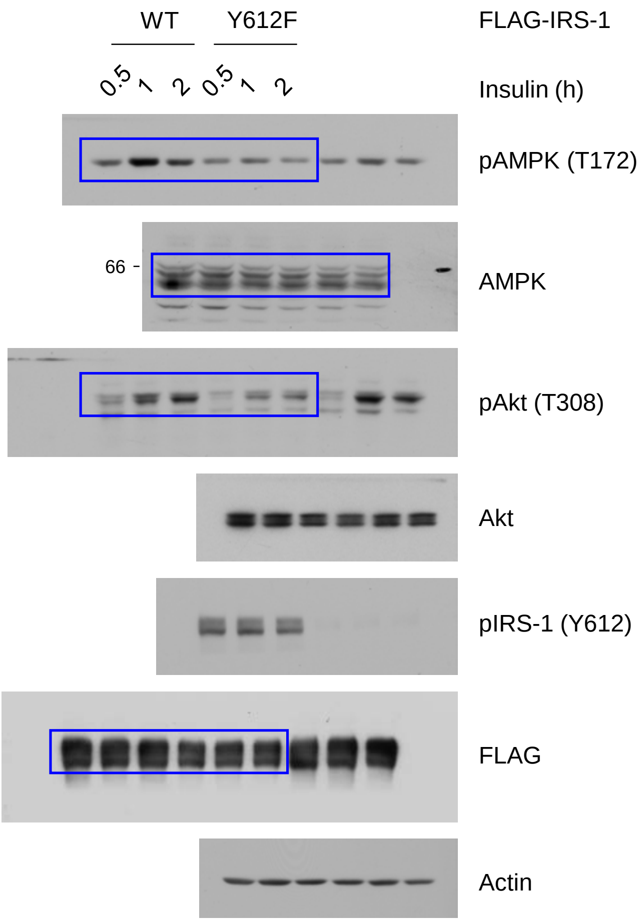

Fig 5B

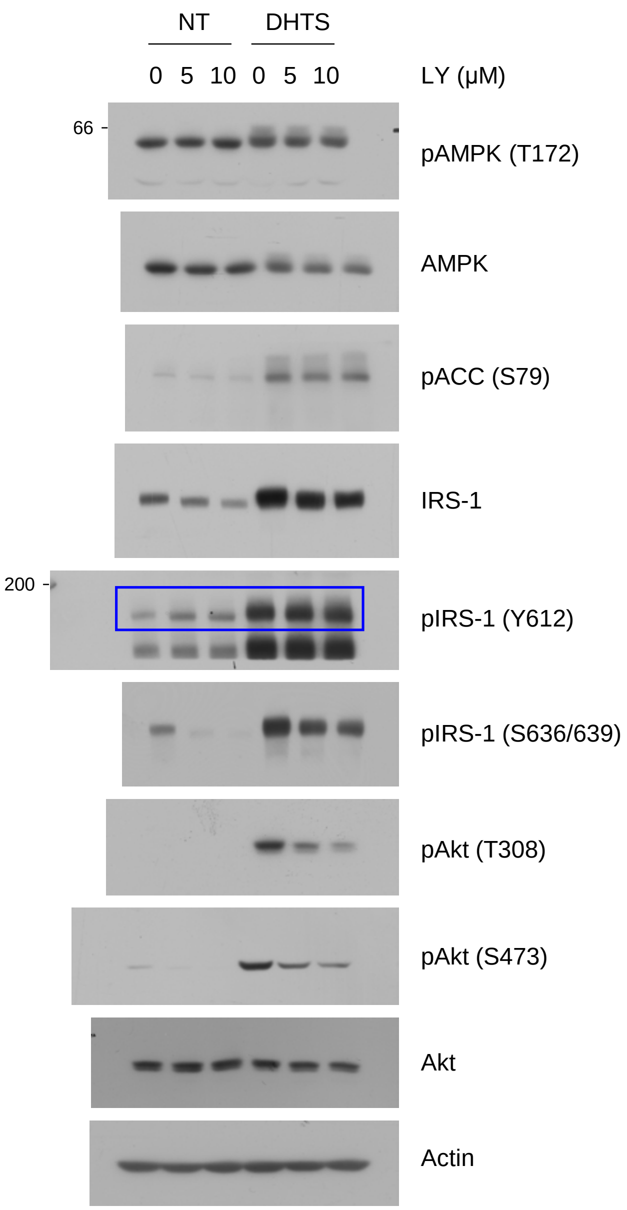

Figure S1. Identification of the C1-Ten inhibitor.

(A) Purification of active C1-Ten protein. Quantity, integrity, and purity of the proteins were determined by Ponceau S staining (left). PTPase activity of purified C1-Ten protein was measured each time prior to *in vitro* screening (right).

(B) Names and structures of the naphthoquinone derivatives.

(C) HEK293 cells were transfected with FLAG-C1-Ten and HA-IRS-1. Immunoprecipitation was performed with anti-FLAG antibody in the presence of DHTS (0, 20, or 50 μM).

Figure S2. DHTS increases glucose uptake in dexamethasone-induced insulin resistant conditions.

(A) Dexamethasone-induced insulin resistance. L6 myotubes on day 9 were incubated with 200 nM dexamethasone (Dex) for 24 h. The cells were serum starved for 2 h and stimulated with 10 nM insulin for 20 min.

(B) Effect of DHTS on glucose uptake in myotubes. Dexamethasone-treated L6 myotubes were incubated with 10 μM DHTS for the times indicated, and 2-deoxy[^{14}C] glucose was measured. Data are presented as the mean ± SEM of three biological replicates, and similar results were obtained from three independent experiments.

(C) Effect of DHTS on *ex vivo* glucose uptake. Prepared primary EDL cells were incubated with DHTS (10 μM) for 1 h in the absence or presence of insulin (100 nM, 15 min) and assayed for glucose uptake. Data are presented as the mean ± SEM (n = 3); *$P < 0.05$ and ***$P < 0.001$.

Figure S3. DHTS increases the IRS-1 protein, but not the mRNA level.

(A) Effect of C1-Ten knockdown on DHTS-induced IRS-1 recovery in L6 myotubes. L6 myoblasts were transfected with 100 nM of control or C1-Ten siRNA at 24 h post-seeding. Transfected myoblasts were induced to differentiate into myotubes.
On day 8, the cells were treated with DMSO or 5 μM DHTS for 30 min.

(B) IRS-1 mRNA levels after DHTS treatment. L6 myotubes were incubated with 200 nM dexamethasone for 24 h, followed by treatment with 10 μM DHTS for 1 h.

Figure S4. DHTS activates AMPK in LKB1 and CaMKK independent manner.

(A) HeLa cells were incubated with 20 μM DHTS for 1 h.

(B) After pre-treatment with 2.5 μM STO609 (STO) for 30 min, L6 myotubes were co-treated with STO and 10 μM DHTS for 1 h. Data are presented as the mean ± SEM (n = 4); **$P < 0.01$; NS, not significant.

(C) Soleus muscles from 5- to 6-week-old C57BL/6J mice were incubated with medium containing vehicle (DMSO) or 20 μM DHTS for 2 h. Data are presented as the mean ± SEM (n = 10); *$P < 0.05$ and **$P < 0.01$.

Figure S5. Uncropped images of Western blots.
